# Transcriptomic and Proteomic Analyses of Resistant Host Responses in *Arachis diogoi* Challenged with Late Leaf Spot Pathogen, *Phaeoisariopsis personata*

**DOI:** 10.1371/journal.pone.0117559

**Published:** 2015-02-03

**Authors:** Dilip Kumar, Pulugurtha Bharadwaja Kirti

**Affiliations:** Department of Plant Sciences, School of Life Science, University of Hyderabad, Hyderabad, India; Università della Calabria, ITALY

## Abstract

Late leaf spot is a serious disease of peanut caused by the imperfect fungus, *Phaeoisariopsis personata*. Wild diploid species, *Arachis diogoi*. is reported to be highly resistant to this disease and asymptomatic. The objective of this study is to investigate the molecular responses of the wild peanut challenged with the late leaf spot pathogen using cDNA-AFLP and 2D proteomic study. A total of 233 reliable, differentially expressed genes were identified in *Arachis diogoi*. About one third of the TDFs exhibit no significant similarity with the known sequences in the data bases. Expressed sequence tag data showed that the characterized genes are involved in conferring resistance in the wild peanut to the pathogen challenge. Several genes for proteins involved in cell wall strengthening, hypersensitive cell death and resistance related proteins have been identified. Genes identified for other proteins appear to function in metabolism, signal transduction and defence. Nineteen TDFs based on the homology analysis of genes associated with defence, signal transduction and metabolism were further validated by quantitative real time PCR (qRT-PCR) analyses in resistant wild species in comparison with a susceptible peanut genotype in time course experiments. The proteins corresponding to six TDFs were differentially expressed at protein level also. Differentially expressed TDFs and proteins in wild peanut indicate its defence mechanism upon pathogen challenge and provide initial breakthrough of genes possibly involved in recognition events and early signalling responses to combat the pathogen through subsequent development of resistivity. This is the first attempt to elucidate the molecular basis of the response of the resistant genotype to the late leaf spot pathogen, and its defence mechanism.

## Introduction

Plants have their own surveillance system to recognize attacking microorganisms and to induce effective defence mechanisms. However, diminished encounter mechanism in the host results in microbial invasion causing deleterious effects, such as diversion of nutrients, metabolites and toxin production, which enhance the disease progression and subsequently death of the tissue. Defence responses are frequently controlled by interaction between plant resistance (R) genes and pathogen avirulence (avr) genes through gene-for-gene interaction [1–3]. Plant-pathogen interaction studies have the immense commercial importance as pathogen attack can lead to massive yield penalties in the concerned crop. Understanding compatible and incompatible plant-pathogen interaction mechanism by which plants resist infection or susceptible to microbial pathogen is very important from an agricultural standpoint.

Defence responses include cell wall fortification, defence related responses such as hypersensitive response [2,4], production of reactive oxygen species [5], production of antimicrobial compounds [6], pathogenesis related protein [7] followed by production of phytoalexins and secondary metabolites. Peanut, *Arachis hypogaea* is one of the most important oil seed crops in the world, particularly in the Semi Arid Tropical region and is widely cultivated for its high quality edible oil and high protein content in the seed. Because of the rain-fed nature of the crop, its yields depend on the vagaries of nature in the form of biotic and abiotic stresses. Biotic stresses include diseases caused by fungal pathogens such as early leaf spots caused by *Cercospora arachidicola* and late leaf spot caused by *Phaeoisariopsis personata* (Berk & Curtis, previously known as *Cercospora personata*) and the rust caused by *Puccinia arachidis*. Late leaf spot disease is the most devastating of these and can lead to yield losses up to 70% under favourable conditions [8].

The peanut genome (2,800 Mb/1C) is large [9] in comparison to other plant models, *Arabidopsis* (128 Mb), rice (420 Mb), *Medicago* (500 Mb) and soybean (1,100 Mb). However, the peanut research community across the globe submitted nearly 252,832 expressed sequence tags (ESTs) in the public NCBI database till March 2012 in comparison to closely related soybean, which is represented by 1,461,624 ESTs [10]. It has also been reported that an analysis of the peanut transcriptome by RNA-seq using next-generation Illumina sequencing during seed development has generated a large number of unigenes and about four thousand SSR primers from three different varieties of peanut [11]. Guo, et al. [12] constructed cDNA libraries for peanut gene expression profiling in developing seeds at different reproductive stages during *Aspergillus parasiticus* infection. Despite these transcriptome analyses, there were no reports on the availability of sources of disease resistance genes in the cultivated genotypes of peanut. However, wild relatives of the Genus *Arachis* are a rich source of genes for disease resistance, which can be exploited by cloning through genomic approaches. In the genus *Arachis*, there are many wild species at diploid and allo-tetraploid levels that possess resistance to various biotic and abiotic stresses making them a rich repository of genes of commercial importance. There were many attempts aimed at transferring the genes for disease resistance from the wild species to the cultivated accessions through conventional breeding programs [13,14]. However, these attempts proved to be unsuccessful as the introgression of genes from these wild species also resulted in a linkage drag transferring unnecessary gene blocks carrying the desired genes, and making the introgressed material unsuitable for use in subsequent breeding programs.

Strategy to improve resistance is to characterize and clone novel resistance gene homologs from the resistant wild relatives. Several diploid wild species of the genus *Arachis*, Viz., *A*. *diogoi*, *A*. *stenosperma*, *A*. *cardenasii*, *A*. *duranensis* etc. show very high levels of resistance to fungal and rust pathogens [15]. These will constitute ideal material to study the differences at molecular level involved in conferring resistance or susceptibility. Nobile, et al. [16] group elucidated the defence strategies of peanut by using the approach of suppression subtractive hybridization and Guo, et al. [17] used cDNA microarray strategy to identify the gene (s) for resistance to *Aspergillus flavus* in peanut. Payton, et al. [18] compared gene expression profile in a variety of peanut plant tissues using high density oligonucleotide microarrays. Recently, a study on the differential gene expression in *Arachis diogoi* upon infection from the late leaf spot pathogen was reported by using Genefishing DEG premix kit in a differential display-reverse transcription PCR study [19] and differentially expressed peanut genes were identified and analyzed in response to challenge with bacterial wilt disease caused by *Ralstonia solanacearum* [20]. Peng, et al. [21] identified 119 TDFs from resistant and susceptible cultivars of peanut (Spanish type) using cDNA-AFLP after inoculation with the bacterial pathogen, *Ralstonia solanacearum* that causes wilt disease and studied their expression patterns.

Several methods are available for studying differential gene expression and cDNA-AFLP is an extremely efficient, sensitive and reproducible technique for the detection of differentially expressed genes [22,23]. It is a genome-wide expression analysis technique, which does not require prior sequence information, which makes it an excellent tool for gene discovery [24]. In relation to hybridization-based techniques, such as macro- and microarrays, cDNA-AFLP can discriminate between homologous genes belonging to gene families that are very common in plants. Besides, the sensitivity of the technique is very high resulting in an excellent detection of low-abundance transcripts and, both induced and repressed genes can be easily detected [25]. There are many examples of the successful use of the cDNA-AFLP as a genome-wide expression analysis tool of genes involved in various biological processes ranging from plant development to responses to environmental stimuli. Wang, et al. [26,27] revealed differential gene expression in incompatible and compatible interaction of wheat challenged with stripe rust fungus using cDNA-AFLP while Cheng, et al. [28] identified differentially expressed genes induced by bamboo mosaic virus infection in *Nicotiana benthamiana* by the same technique. Studies on abiotic stresses like response to salt in a halophyte, *Spartina alterniflora* [29], drought stress in *Popolus hopeiensis* [30] and heat stress in rice [31] also revealed differentially expressed genes in transcriptome profiling by cDNA-AFLP leading to the identification of the candidate genes.

Proteomic analysis reveals the translational products of gene expression of plant under stress condition and its physiological state under particular conditions. Analysis of proteins is a direct approach to define the function of their associated genes as it linked to genome sequence information, which is important for functional genomics. There are scanty reports of proteome analysis that focuses on the study of stress response of peanut genotypes against various stress conditions. Wang, et al. [32] analysed peanut seed proteins differentially expressed in resistance and susceptible peanut cultivars in response to *Aspergillus flavus* and reported expression of several disease resistance associated protein. Proteomic study of peanut cotyledons in response to atoxigenic and toxigenic *A*. *flavus* strains revealed aflatoxin-triggered immune response [33]. Kottapalli, et al. [34,35] have analysed seed proteins of four peanut cultivars, which revealed differential expression of storage, allergenic proteins and also identified several physiologically significant candidate proteins associated with water-deficit stress tolerance mechanism in three peanut genotypes. Katam, et al. [36] carried out proteomic study in peanut leaf using a drought-tolerant variety and identified more than 200 proteins, predominantly carbohydrate metabolism and photosynthesis related proteins, which help to understand peanut leaf protein alterations under varied stress conditions.

Differential proteomic study of plant-pathogen interaction successfully reported in several plants. For example, Kaur, et al. [37] identified defence-related proteins, which are required for mounting a successful defence response in *Brassica juncea* against *Albugo candida*. Recently, Wu, et al. [38] analysed a plant-virus interaction in resistant and susceptible ecotypes of maize infected with sugarcane mosaic virus and identified several defence and stress related proteins during both compatible and incompatible interaction. The model plant, *Arabidopsis thaliana* differentially expressed proteins related to oxidative stress and metabolism in response to treatments with fungal elicitors in *Arabidopsis* cell cultures [39]. Castillejo, et al. [40] analysed root pea proteome in response to *Orobanche crenata* inoculation and identified several proteins with protease activity which could play an important role in preventing the pathogen and some of metabolism and stress response protein.

The objective of this study is to investigate the molecular responses of the wild peanut, *Arachis diogoi* when challenged with the fungal pathogen, *Phaeoisariopsis personata* and we have identified genes and proteins differentially expressed during the incompatible interaction in wild peanut by the cDNA-AFLP and 2D gel electrophoresis technique. We have validated the expression patterns of some of the genes and the novelty of this study lies in a comparative analysis of the expression of these selected genes in compatible (susceptible) and incompatible (resistance) interactions at different time points through qRT-PCR. Till date, there is no report of proteome analysis that focuses on study of susceptible and resistant peanut genotypes against late leaf spot disease. The fold changes of differentially expressed proteins upon pathogen challenge were found to be high in case of *A*. *diogoi* (resistant) in comparison to *A*. *hypogaea* (susceptible). Here, we report a number of gene fragments that were found to be induced or repressed during incompatible interaction between the wild peanut and *P*. *personata*, and the proteins involved in signalling, metabolism, defence responses as well photosynthesis were found differentially expressed. Differentially expressed genes and proteins were discussed with emphasis on their involvement in defence, signal transduction and others cellular metabolic processes. These observations may possibly reveal genes and proteins, which might be useful in allowing the host plant to cope up with the invading pathogen and provide new insights into the molecular mechanism of plant-fungal interaction.

## Materials and Methods

### Plant material

Wild species, *Arachis diogoi* (accession number ICG-8962) supplied by the International Crops Research Institute for Semi Arid Tropics, Patancheru, India was used for the cDNA-AFLP and proteomics analysis of an incompatible interaction. For the qRT- PCR analysis of some candidate genes, peanut (*Arachis hypogaea*) cultivar cv. JL-24 (susceptible) was used in comparison to the wild species to validate some of the genes identified in the cDNA-AFLP analysis. Plants were maintained under greenhouse conditions. Shoots from wild and cultivated materials were detached from 60 day old plants with a sterile blade, washed thoroughly with sterile distilled water and maintained in plastic trays on moist filter paper with ends of the cuttings wrapped in water-soaked cotton. Trays were covered with polythene sheets to maintain high humidity and kept in growth room at 25±1°C with a photoperiod of 14 h of light and 10 h of dark to enable the cuttings to get stabilized/ acclimatized till the formation of adventitious roots at the cut ends.

### Experimental treatments

Initially, the germinability of the conidia of *Phaeoisariopsis personata* was checked and the inoculum was prepared by suspending the conidia at a concentration of 10^5^/ml in 0.02% Tween-20 and spread on the abaxial surface of leaves homogeneously by using a paint brush and the rooted twigs were maintained under high humidity in the growth room. Control material corresponding to each time point was brushed with 0.02% Tween-20. Leaf tissues of treated and controls of both plant species, *Arachis diogoi* (resistant) and *Arachis hypogaea* cv. JL-24 (susceptible) were harvested at 0, 24, 48, 72 and 96 hours post inoculation and quickly frozen in liquid nitrogen, and stored at -80°C prior to total RNA and protein extraction. Plants were observed for symptom development 20 days post inoculation (S1 Fig.).

### RNA extraction and double stranded cDNA synthesis

Total RNA from control and treated, *P*. *personata* infected leaves was extracted according to method of Chang, et al. [41]. To avoid DNA contamination, total RNA was treated with RNase free DNase1 (Sigma-Aldrich, USA) according to the manufacturer’s instructions. The quality of RNA was checked using a spectrophotometer (NanoDrop Technologies Inc., USA) at two wavelength ratios of A260/230 and A260/280 nm. The integrity of total RNA was determined by running samples on ethidium bromide stained 1.2% agarose gels using Tris boric acid EDTA (TBE) buffer. For cDNA synthesis 2 μg of total RNA from each sample was used for first strand synthesis followed by second strand synthesis using superscript double stranded cDNA synthesis kit (Invitrogen, Carlsbad, USA) as per the manufacturer’s instructions.

### cDNA—AFLP analysis

About 500 ng double stranded cDNA was used for standard AFLP template according to Vos, et al. [22] and Bachem, et al. [23]. cDNA-AFLP was performed using the AFLP core reagent kit and the AFLP PreAmp primer mix-1 (Invitrogen, Life Technologies, Merelbeke, Belgium). In brief, the double stranded cDNA samples were digested with the restriction enzymes, *Eco*RI (Rare cutter) and *Mse*I (Frequent cutter). The digested products of cDNA were ligated to adapters as provided in the kit and the sequences of these adapters are as follows: *Eco*RI adapter, 5′- CTCGTAGACTGCGTACC-3′ and 3′-CTGACGCATGGTTAA-5′, *Mse*I adapter, 5′-GACGATGAGTCCTGAG-3′ and 3′-TACTCAGGACTCAT-5′. The ligation products were then pre-amplified using *Eco*RI 5′-GACTGCGTACCAATTC-3′ and *Mse*I 5′-GATGAGTCCTGAGTAA-3′ pre-amplification primers corresponding to the *Eco*RI and *Mse*I adapters with one base pair extension. Pre-amplification was performed for 20 cycles at 94°C for 30 sec, 56°C for 60 sec and 72°C for 60 sec followed by 5 min at 72°C. Specific amplification was done using pre-amplified products as template with one primer labelled with Cyanine-5 (Cy5) fluorescent dye. Selective amplification primers have been provided with selective nucleotides at their 3′ ends. The primers for this study are as follows: *Eco*RI E-AAG, E-AGC, E-ACG, E-AAC, E-ACA, E-ACT, E-ACC & E-AGG; and *Mse*I M-CAA, M-CAC, M-CAG, M-CAT, M-CTA, M-CTC, M-CTG & M-CTT, generating a total of 64 different combinations. *Eco*RI series (E-series) primers were labelled with fluorescent dye Cy5 for visualization of the bands upon fluorescent scanning. Selective *Mse*I primer and fluorescent (Cy5) labelled *Eco*RI primer combinations were then subjected to amplification of pre-amplified cDNA in thermal cycler following touchdown PCR conditions; 2 min denaturation at 94°C, followed by 30 s denaturation at 94°C, 30 s annealing at 65°C, 60 s extension at 72°C (13 cycles, scale down of 0.7°C per cycle); 30 s denaturation at 94°C, 30 s annealing at 56°C, 60 s extension at 72°C (23 cycles) and 7 min at 72°C. Six microlitre volume of the amplified products were mixed with equal volume of loading buffer (Bromophenol Blue in 25 mM EDTA and 1.5 mM formamide 1:5), heat denatured and resolved on a 8 M urea/6% denaturing polyacrylamide sequencing gel run with 1X TBE electrophoresis buffer at 110 watts. One of the gel glass plates was treated with the bind Silane M-6514 (Sigma-Aldrich) to stick the gel while sigma cote (Sigma-Aldrich) was applied on the second glass plate to repel the gel. The resulting glass-backed polyacrylamide gel has been used to perform silver staining for the visualization of the amplification products.

### Fluorescence scanning, silver staining and isolation of transcript-derived fragments (TDFs)

Sandwich gel was scanned on Typhoon imager (Typhoon Trio and Typhoon 9410) for the detection of bands having Cy5-labelled primers applying a laser excitation at 633 nm and an emission filter at 670 nm (S2 Fig.). Then, the gel was silver stained in a 40 X 30 cm plastic tray on a shaker as described by Creste, et al. [42] with modifications. Silver stained and visualized differentially expressed TDFs were excised from the gel with a surgical blade and eluted in 100 μl of sterile double distilled water. They were heated at 95°C for 15 min and then, hydrated overnight at 4°C according to Baisakh, et al. [29]. Five μl of eluted DNA was used for re-amplification by using same primer combination and identical PCR conditions. The PCR products were examined on a 1.2% agarose gel and visualized on an UV transilluminator using ethidium bromide stain. The products were further purified using gel extraction kit (Sigma-Aldrich, USA).

### Cloning and sequence analysis of TDFs

The eluted amplicons were cloned into the pTZ57R/T cloning vector (Fermentas, Germany) and sequenced commercially. Sequence information of cloned TDFs was analysed by searching for homologous sequences in non-redundant and EST databases of NCBI using basic local alignment search tools (Blastn and Blastx). Annotation was based on the best match found in blastx alignment. The putative functions of the identified genes were assigned based on their similarity with other genes available in the database. The sequences were submitted to genbank (NCBI) and the accession numbers were released in public database (S3 Table).

### Total protein extraction and two dimensional gel electrophoresis (2D-GE)

Proteins were extracted from the sampled leaf tissues by phenol extraction method as described by Sarvanan and Rose [43] with some modifications. One gram of groundnut leaf tissues were ground suspended in 10 ml of the extraction buffer (0.5 M Tris—HCl at pH 7.5, 0.7 M sucrose, 0.1 M KCl, 50 mM EDTA, 2% β-mercaptoethanol and 1 mM PMSF). Equal volume of phenol saturated with Tris-HCl (pH 7.5) was added, mixed for 30 min at 4°C and centrifuged at 6,000 g for 25 min at 4°C. The upper phenolic phase was collected and an equal volume of extraction buffer was added to it and this step was repeated again. Four volumes of 0.1 M ammonium acetate in methanol was added and kept overnight at -20°C for protein precipitation. The samples were then centrifuged at 12,000 g at 4°C for 20 min and the precipitate was washed three times each in ice cold methanol as well as in ice cold acetone and air dried. The precipitates were resuspended in 500 μl of rehydration buffer [7 M (w/v) urea, 2 M (w/v) thiourea, 4% (w/v) CHAPS, 30 mM DTT, 0.8% (v/v) immobilized pH gradient (IPG) buffer pH range 4–7 (GE Healthcare, Uppsala, Sweden)], insoluble material was removed by centrifugation and the protein concentration was estimated by Bradford assay using BSA as a standard.

800 μg of total protein in rehydration buffer in a total volume of 320 μl was used for passive rehydration of 18 cm immobilized pH gradient (IPG) strips (18 cm, 4–7 pH linear gradient; Amersham, GE). Active rehydration of protein was done on immobilized pH gradient strips for 12 h at 50 V. Rehydration and focusing was carried out in Ettan IPGphor II (GE Healthcare) at 20°C, using the following program: 30 minutes at 500 V, 3 h to increase from 500 to 10,000 V and 6 h at10,000 V (a total of 60,000 Vh). After IEF, strips were equilibrated twice to reduce the protein followed by alkylation for 25 minutes with gentle rocking at room temperature (25 ± 2°C) in the equilibration buffers using 2% (w/v) DTT and 2.5% (w/v) iodoacetamide respectively. The proteins were separated in the second dimension SDS-PAGE (12% vertical polyacrylamide slab gels) at 10 mA gel¯^1^ for 1 h and then 38 mA gel¯^1^ for 6 h, using an EttanDalt6 chamber (GE Healthcare). Gels were stained with modified Coomassie staining [44]. Protein gels were scanned by a calibrated densitometric scanner (GE Healthcare) and spot detection, normalization, gel matching, expression analyses and statistics were conducted with Image Master 2D Platinum v. 6.0 image analysis software (Amersham Biosciences). Proteins that displayed one and half fold or greater changes in the relative spot volume were considered as altered expressed proteins.

### In gel digestion and mass spectrometry (MS)

Interested protein spots were excised from three coomassie-blue stained replicated gels and destained with 200 μL of 50% acetonitrile (ACN) in 50 mM of ammonium bicarbonate (NH_4_HCO_3_) until completely destained. Thereafter, the gel pieces were treated with 10 mM DTT in 50 mM NH_4_HCO_3_ and incubated at 56°C for 1 h. This was followed by treatment with 55 mM iodoacetamide in 50 mM NH_4_HCO_3_ for 45 min in dark at room with temperature at 25 ± 2°C. The gel pieces were then washed with 25 mM NH_4_HCO_3_ and ACN, dried in Speed Vac at ambient temperature and rehydrated in 15 μl of 25 mM NH_4_HCO_3_ solution containing 25 ng μl^-1^ trypsin at 4°C for 10 minutes and then digested at 37°C overnight (sequencing grade, Promega Corporation, Wisconsin, USA). After incubation, a short spin was given and the supernatant was collected in a fresh eppendorf tube. The left gel pieces were further sonicated for 10 minutes followed by frequent vortexing for 5 min in 10 μl of 0.1% trifluoroacetic acid (TFA) and 100% ACN (1:1) to extract the remaining peptides. This extraction step was repeated twice to improve the extraction yield. The supernatants were pooled together and dried using Speed Vac and were reconstituted in 5 μl of 100% ACN and 0.1% TFA (1:1 v/v). The above sample (1 μl) was mixed with 1 μl of a cyano-4-hydroxycinnamic acid (CHCA) matrix in 50% ACN and 1% TFA (1:1) and 2 μl of samples were spotted onto a MALDI plate and dried at room temperature for mass spectrophotometry. Matrix-assisted laser desorption/ionization time of flight mass spectrometric (MALDI-TOF MS) analysis was carried out using MALDI-TOF/TOF mass spectrometer (Bruker Autoflex III Smartbeam, Bruker Daltonics, Bremen, Germany) according to the protocol of Shevchenko, et al. [45] with minor modifications. Mass data acquisitions were piloted by FlexControl 3.0 (Build 100) software using batched-processing and automatic switching between MS and MS/MS modes. Peptide precursor ions corresponding to contaminants including keratins and trypsins autolytic products were excluded in a mass tolerance of ± 0.5 Da. The filtered precursor ions with a user defined threshold were selected for the MS/MS scan.

### Protein identification through peptide mass fingerprinting and MS/MS analysis

The MALDI-TOF/TOF data were loaded into the MASCOT program (http://www.matrixscience.com) employing Biotools software (Bruker Daltonics) and protein identification was performed against the NCBInr and Swiss-Prot databases using a combination of MS (peptide mass fingerprint approach) with MS/MS. The taxonomic category was set to *Viridiplantae* (Green plants). The other search parameters were: monoisotopic peptide mass (MH^+^); one missed cleavage per peptide; enzyme, trypsin; precursor-ion mass tolerance on an average 200 ppm; MS/MS fragment-ion mass tolerance, 0.6 Da; variable modifications like carbamidomethylation (C) for cysteine and oxidation for methionine (M) were allowed. If a protein spot matched multiple proteins under different accession numbers, the candidate protein with the maximum Mascot score were selected. The nearest experimental MW (molecular weight) and PI (isoelectric point) values to the theoretical values (having the same Mascot score) were given equal weightage in spot selection. The identified proteins were named according to the corresponding annotations in NCBI and Swiss-Prot.

### Quantitative real time PCR analysis

qRT-PCR was carried out for nineteen selected gene fragments at different time points after treatment with the fungal pathogen to validate the differential gene expression data obtained from cDNA-AFLP analysis. Leaf tissues of resistant (*Arachis diogoi*) and susceptible (*Arachis hypogaea*) plant materials challenged with *Phaeoisariopsis personata* and the samples were collected at 0, 24, 48, 72, 96 hpi (hour post inoculation) as well as mock inoculated plants. Gene specific primers were designed for 19 TDFs (S1 Table) chosen for validation, using oligo analyzer software (IDT). The total RNA (2 μg) treated with DNase I was reverse transcribed to first strand cDNA with oligo dT (18 mer) and random hexamer primer for Alcohol dehydrogense-3 and 60S ribosomal protein respectively, using SMART MMLV Reverse Transcriptase (Clontech, Becton Dickinson, USA). First strand cDNA samples were diluted 10 times and 1μl of the diluted reaction mixture was taken as qRT-PCR template in a 20 μl total reaction volume containing 0.4 μM gene-specific primers and 10 μl SYBR Premix Ex Taq with ROX (TAKARA BIO INC.) and the samples were appraised in three technical replicates including three non-template as the negative control. PCR analysis was carried out in Realplex (Eppendorf, Germany) Amplifier with the following cycle parameters: 95°C for 5 min; 40 cycle of 95°C for 20 s, 58°C for 20 s, 72°C for 20 s followed by melting curve to ensure that each amplicon was a single product. Alcohol dehydrogenase class III (*adh3*) and 60S ribosomal protein genes were used as internal control for calculating relative quantification of gene expression as these are the most stable reference gene for *Arachis* to normalize the real time amplification data [46]. Relative fold change in RNA expression was estimated using threshold cycle (C_T_) to calculate the relative fold change (RFC) in each time point of infected sample compared to control conditions by ΔΔC_T_ method [47].

## Results

### Late leaf spot infection analysis in peanut leaves of resistant and susceptible plants

We have investigated pathogenesis pattern of *P*. *personata* in peanut during infection by light microscope and found that leaf spot disease appeared on the susceptible host plant (*Arachis hypogaea* cv. JL-24) after 10–12 days post inoculation of fungal conidia, while no such disease symptoms were found on the resistant wild species (*Arachis diogoi*). An early stage was chosen for analysis, as the conidia of *P*. *personata* were observed to germinate after 12–24 hpi (S1 Fig.) and their germ tubes enter the plant cells directly via the epidermis or more frequently through stomata, allowing the intracellular mycelial growth [48,49]. Spots were fully developed after 20–24 days post inoculation in compatible interaction, but no such lesions were observed in incompatible interaction (S1 Fig.). We chose to indentify changes in gene and protein expression analysis in incompatible and compatible interaction at early stage of infection.

### Identification of differentially expressed transcripts during *A*. *diogoi* and *P*. *personata* interaction

We carried out a cDNA-AFLP analysis on the RNA samples of *Arachis diogoi* a wild accession as control and treated samples, which were pooled from different stages such as 0, 24, 48, 72, 96 hpi of infection with *P*. *personata* along with a mock treatment. A total 64 primer combinations were used to visualize 4047 TDFs in control and treated samples. The number of amplified fragments varied from 30 to 55 per lane and their sizes ranged from 75 to 700 bp depending upon primer combinations and a representative gel picture was illustrated in S2 Fig. A total 233 differentially expressed gene fragments were selected on the basis of their intensity differences between control and treated sample, of which 125 were upregulated, 64 downregulated and 44 were point expressed, indicating that genes were expressed during interaction and the point expressed TDFs were classified under upregulated gene fragments. These selected TDFs were recovered from gels, re-amplified, sub-cloned and sequenced commercially.

### Gene sequence analysis

The sequences of the 233 transcript derived fragments were annotated by similarity search using the basic local alignment search tool (BLASTX & BLASTN) program against the non-redundant (nr) public database of the NCBI-GenBank. About half of the TDFs were identified to be coding for hypothetical proteins with no significant similarity to existing sequences in the GenBank. This shows the importance of the detailed analyses of the hypothetical proteins in identifying novel genes involved in the tolerance to biotic and abiotic stresses.

**Fig 1:**
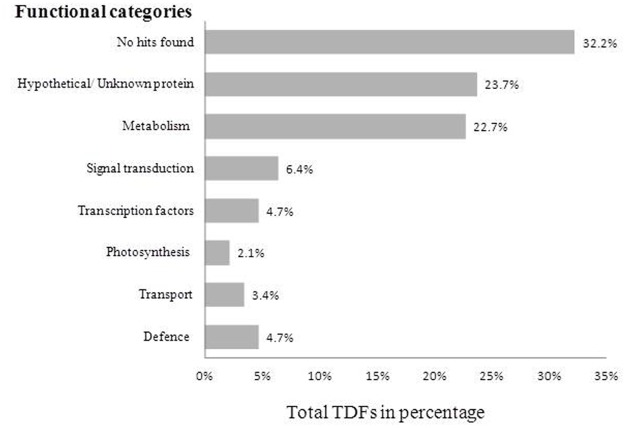
Classification of differentially expressed transcripts (TDFs). A total of 233 TDFs were classified based on the Blastx homology search.

According to Bevan’s method [50], the TDFs were grouped into functional categories based on their homology to known proteins. A major group of 75 sequences (32.2%) showed no significant similarity, while 55 (23.6%) sequences were designated as unknown/hypothetical proteins. Genes involved in metabolism were found to be 53 (22.7%) and 15 (6.4%) sequences shared high similarity with genes functioning in signal transduction. The genes involved in defence and transcription factors shared equal number of 11 (4.7%) sequences, while the rest of the sequences were a group of genes involved in photosynthesis 5 (2.2%) and transport 8 (3.4%) (Fig. 1). Differentially expressed TDFs were identified on the basis of band intensity between control and treated samples. In comparison to control with high band intensity were considered as up-regulated while low intensity bands were grouped as down-regulated. The differentially expressed upregulated, downregulated TDFs were listed in Table 1 and their sequences were submitted to NCBI database with assigned accession numbers. Most of the sequences matched with the ESTs reported for *Glycine max* and *Medicago truncatula* with significant similarity.

**Table 1:** Important gene fragments and their significant similarity; U- Upregulated, D- Downregulated.

TDF No.	Accession	Length	U/D	Annotation (BlastX), Organism	E- value
				Signal transduction	
1.	GU011970	309	D	Zinc finger protein, putative [*Ricinus communis*]	6e-44
2.	FJ581437	541	D	Receptor kinase, putative [*Ricinus communis*]	6e-99
3.	GU320766	120	U	DNA-binding SAP; Zinc finger, MIZ-type; Zinc finger, FYVE/PHD-type [*Medicago truncatula*]	2e-07
4.	GQ922055	358	U	Serine/threonine-protein kinase PBS1, putative [*Ricinus communis*]	7e-58
5.	GU592825	231	U	Protein kinase (PK) [*Fagus sylvatica*]	2e-23
6.	GU592827	420	D	DNAJ heat shock N-terminal domain-containing protein [*A*. *thaliana*]	1e-68
7.	GU133626	243	U	Chaperone protein DnaJ-like [*Glycine max*]	6e-06
8.	GU326970	126	U	F-box family protein [*Populus trichocarpa*]	6e-06
9.	GU062406	213	U	Sister chromatid cohesion 1 protein, putative [*Ricinus communis*]	1e-18
				Defence	
10.	EU935215	104	U	Cystatin [*Spinacia oleracea*]	9e-20
11.	GQ922057	351	U	SGT1–2 [*Glycine max*]	6e-53
12.	GQ922059	429	U	Heat shock 70 kDa protein, mitochondrial-like [*Glycine max*]	6e-89
13.	GU592820	351	U	CC-NB-LRR type disease resistance protein Rps1-k-2 [*Glycine max*]	4e-34
14.	GQ466607	666	U	Thaumatin-like protein 1a-like [*Glycine max*]	7e-93
15.	JN160607	240	U	Vacuolar-processing enzyme-like [*Glycine max*]	9e-41
16.	FJ581436	298	U	rac GTPase activating protein 1 [*Lotus japonicus*]	2e-24
17.	GU785018	285	U	NADPH oxidoreductase/15- Hydroxyprostaglandin dehydrogenase [*Medicago truncatula*]	1e-47
				Metabolism	
18.	GU223572	408	U	Isoamyl acetate-hydrolyzing esterase, putative [*Ricinus communis*]	1e-57
19.	GU223575	201	U	Late embryogenesis abundant protein Lea14-A, putative [*Ricinus communis*]	6e-11
20.	GU223576	142	U	Similar to beta-glucosidases [*Arabidopsis thaliana*]	1e-11
21.	GU011969	440	D	Ribonucleoprotein, chloroplast, putative [*Ricinus communis*]	2e-52
22.	GU223577	288	D	Nucleotide binding protein, putative [*Ricinus communis*]	3e-52
23.	GU011971	150	U	Sedoheptulose-bisphosphatase precursor [*Arabidopsis thaliana*]	2e-28
24.	GU223578	310	U	Exostosin-like [*Medicago truncatula*]	3e-27
25.	EU935216	362	U	Adenosine 5′-phosphosulfate reductase [*Glycine max*]	4e-74
26.	GU320767	115	U	Putative beta-galactosidase [*Glycine max*]	5e-15
27.	GU320768	246	U	Putative mutator sub-class protein [*Arachis hypogaea*]	8e-17
28.	FJ231268	206	U	Methionine synthase [*Glycine max*]	2e-32
29.	GU320771	669	D	Amine oxidase, putative [*Ricinus communis*]	6e-148
30.	FJ621571	498	D	Similar to cysteine protease Cp5 [*Vitis vinifera*]	1e-61
31.	FJ621572	426	D	Polygalacturonase precursor [*Glycine max*]	5e-63
32.	GU326969	261	D	Endo beta n-acetylglucosaminidase, putative [*Ricinus communis*]	6e-12
33.	GU326971	300	U	Polyprotein [*Sorghum bicolor*]	1e-18
34.	GU326972	213	U	Retrotransposon gag protein [*Arachis hypogaea*]	8e-29
35.	GQ922058	432	U	Dihydroflavonol-4-reductase [*Medicago truncatula*]	8e-62
36.	GU473169	288	U	Probable NADH dehydrogenase-like[*Glycine max*]	2e-29
37.	GU473170	267	D	Cellulose synthase catalytic subunit [*Gossypium hirsutum*]	3e-57
38.	GU473171	315	U	Microtubule-associated protein, putative [*Ricinus communis*]	1e-27
39.	GU576547	552	U	GIGANTEA [*Glycine max*]	2e-99
40.	GU576549	285	U	Peroxisomal fatty acid beta-oxidation multifunctional protein [*Glycine max*]	9e-42
41.	GU062405	240	U	Glycine-rich protein [*Arabidopsis thaliana*]	5e-23
42.	GU576554	150	U	Non-phosphorylating glyceraldehyde-3-phosphate dehydrogenase [*Pisum sativum*]	1e-26
43.	GU592818	198	U	Phytochrome A1 [*Glycine max*]	1e-28
44.	GQ979706	226	U	Nucleic acid binding protein, putative [*Ricinus communis*]	4e-47
45.	GU592826	633	D	Granule-bound glycogen (starch) synthase [*Astragalus membranaceus*]	1e-123
46.	GU785014	135	U	N-alpha-acetyltransferase 35, NatC auxiliary subunit-like [*Glycine max*]	3e-15
47.	GU785017	153	U	Short-chain dehydrogenase/reductase [*Medicago truncatula*]	1e-16
48.	FJ226754	338	U	ATP-dependent Clp protease regulatory subunit CLPX [*Arabidopsis thaliana*]	3e-66
49.	JZ356629	210	D	UDP-glycosyltransferase 83A1-like [*Glycine max*]	1e-20
50.	JZ356642	183	U	WD repeat-containing protein 48-like [*Glycine max*]	3e-18
51.	JZ356660	192	U	Tyrosine decarboxylase [*Medicago truncatula*]	9e-08
52.	GU326968	255	U	Gag-pol polyprotein [*Phaseolus vulgaris*]	9e-19
53.	FJ231266	422	D	Multicopper oxidase, putative [*Ricinus communis*]	7e-57
54.	GU576550	168	U	WD-repeat protein, putative [*Ricinus communis*]	4e-16
				Photosynthesis	
55.	EU935214	355	U	Oxygen-evolving complex-related [*Arabidopsis thaliana*]	2e-57
56.	FJ226755	210	U	Photosystem II type I chlorophyll a/b-binding protein[*Glycine max*]	2e-38
57.	GQ979704	187	D	Mg chelatase subunit (46 kD) [*Glycine max*]	1e-18
58.	JZ356640	213	U	Chloroplast magnesium chelatase I subunit [*Pisum sativum*]	3e-13
59.	GQ922056	591	U	Cytochrome P450 monooxygenase CYP97C10 [*Glycine max*]	2e-125
				Transport	
60.	FJ231267	300	D	Nodulin 26-like protein [*Medicago truncatula*]	1e-32
61.	GQ293093	129	D	AAA ATPase; ABC transporter, transmembrane region, type 1 [*Medicago truncatula*]	6e-08
62.	GU062403	204	D	Protein alx, putative [*Ricinus communis*]	2e-17
63.	GU576551	132	U	ATP/ADP transporter [*Populus trichocarpa*]	2e-34
64.	GU062404	130	U	Cytochrome c biogenesis [*Medicago truncatula*]	5e-17
65.	GU592822	147	U	Nucleobase ascorbate transporter [*Populus trichocarpa*]	4e-10
66.	GQ466606	465	D	Glutathione-regulated potassium-efflux system protein kefB, putative [*Ricinus communis*]	2e-63
				Transcription	
67.	GU320773	207	D	ATP-dependent RNA helicase eIF4A, putative [*Phytophthora infestans* T30–4]	5e-35
68.	GU473167	237	U	Squamosa promoter-binding protein, putative [*Ricinus communis*]	1e-19
69.	GU062402	354	D	Valine—tRNA ligase-like protein [*Arabidopsis thaliana*]	5e-67
70.	GU320772	306	U	U3 small nucleolar RNA (U3 snorna) associated protein [*Ricinus communis*]	3e-53
71.	JZ356597	135	U	tRNA dimethylallyltransferase 9-like [*Glycine max*]	8e-15
72.	JZ356611	414	U	RNA-directed DNA polymerase homolog [*Arabidopsis thaliana*]	3e-28
73.	JZ356620	233	U	Reverse transcriptase [*Pisum sativum*]	7e-18

### 2-D gel electrophoresis and protein expression profiling

**Fig 2:**
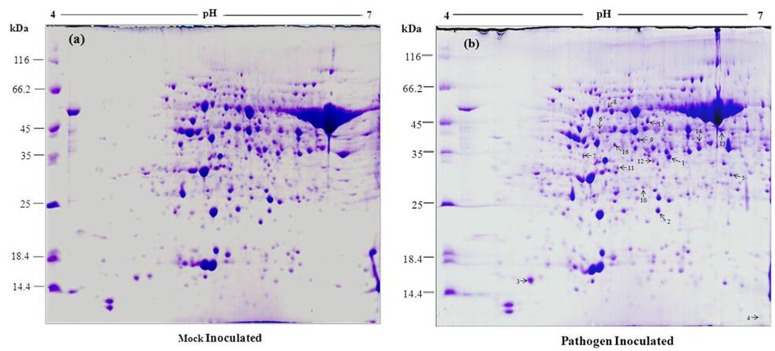
2-DE gel of peanut leaf protein samples from a *Arachis diogoi* (resistant) in response to inoculation with *P*. *personata*, (a) mock inoculated, (b) pathogen inoculated. 800 μg of total leaf protein was loaded on 18 cm IPG strip with a linear gradient of pH 4–7, 12% SDS-PAGE gels were used for second dimension.

**Fig 3:**
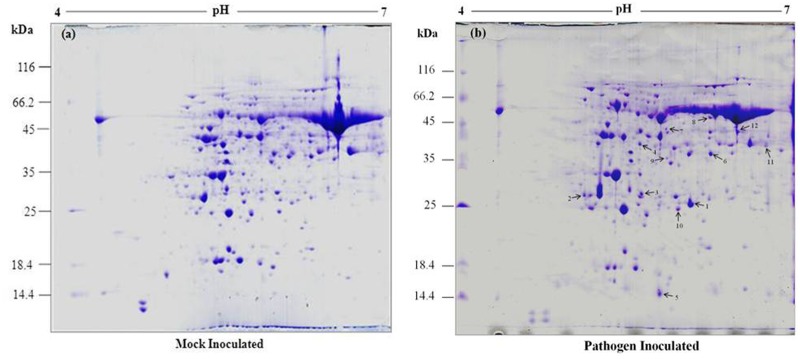
2-DE gel of peanut leaf protein samples from *Arachis hypogaea* L. (susceptible) in response to inoculation with *P*. *personata*, (a) mock inoculated, (b) pathogen inoculated. 800 μg of total leaf protein was loaded on 18 cm IPG strip with a linear gradient of pH 4–7, 12% SDS-PAGE gels were used for second dimension.

Proteomic approach was studied to analyze the changes in protein profile during the interaction between wild and cultivated peanut genotypes and *P*. *personata* as compatible and incompatible interactions. Mostly, resistivity depends upon compatible or incompatible interaction between host and pathogen [51]. Host plants prevent disease development by inducing hypersensitive response in incompatible interaction whereas compatible interaction does not induce HR, results in disease development. In order to understand this mechanism, a comprehensive analysis is required. In this context, a proteomic approach was applied to analyze the changes in protein profile during early stage of peanut and *P*. *personata* interaction. Therefore, leaf samples were selected for proteomic analysis, which were pooled from different stages such as 0, 24, 48, 72, 96 hrs after pathogen inoculation with *P*. *personata* along with a mock treatment. Triplicate gels were obtained from three independent experiments and the representative gels of *Arachis diogoi* (resistant) and *Arachis hypogaea* L. (susceptible) were illustrated in Fig. 2 and Fig. 3 respectively. We observed nearly 350–400 protein spots on susceptible peanut 2-DE gel while 450–500 spots were detected on wild peanut 2-DE gel stained with Coomassie brilliant blue dye. Thereafter, we systematically screened the protein spots that were differentially regulated in response to pathogen challenge using Image Master 2-D platinum version 6 software. There was around 80–85% correlation between biological repeats, indicating reliable reproducibility of the experiments.

**Fig 4:**
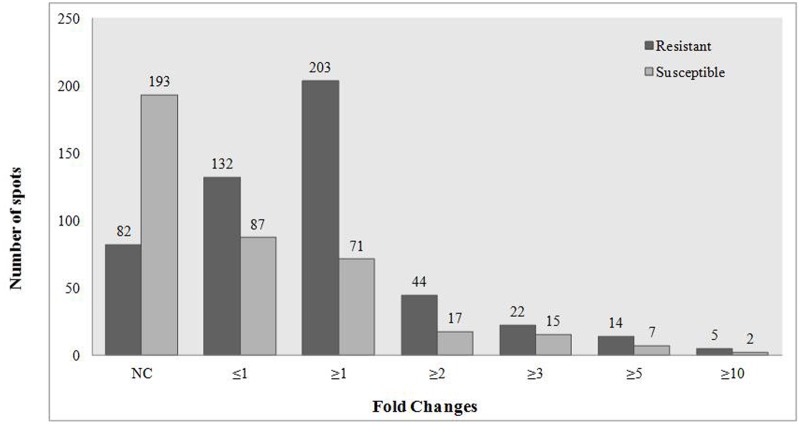
Bar diagram representing fold changes in leaf protein expression pattern in *Arachis diogoi* (Resistant) and *Arachis hypogaea* L. (Susceptible) upon pathogen challenge. NC- no change.

Proteomic study indicated differential expression of proteins both in *A*. *diogoi* and *A*. *hypogaea* upon pathogen challenge. The fold change of differentially expressed proteins was found to be high in case of *A*. *diogoi* in comparison to *A*. *hypogaea* (Fig. 4). In treated *A*. *diogoi*, there were around 44 protein spots observed more than two-fold change, while in *A*. *hypogaea* only 17 protein spots were found more than two-fold expression. There were 22 and 14 protein spots depicted with more than three- and five-fold higher expression respectively in treated *A*. *diogoi* in comparison to susceptible, where as 15 and 7 spots were found similar expression. Five protein spots were detected more than ten-fold expression in *A*. *diogoi*, while only two spots were found with such enhanced changes in *A*. *hypogaea* L. The fold change profiling of proteins are illustrated in Fig. 4. Regulated proteins were determined based on the two basic criterion, reproducibility and fold change (% volume) at least ≥ 1.5 times.

A total of 45 up-regulated and novel protein spots (26 spots from *Arachis diogoi* and 19 spots from *Arachis hypogaea* L.) were selected with reliable expression pattern in peanut and *P*. *personata* interaction for MALDI TOF-TOF analysis. Among 45 spots, only 28 proteins were successfully identified with putative function (Table 2), while the rest 17 did not show any significant hits either in the NCBI or Swiss-Prot databases and hence, were not considered. Out of 28 differentially expressed proteins, 16 identified proteins were found to be up-regulated in *Arachis diogoi*, whereas 12 protein spots were found up-accumulated in *Arachis hypogaea* L. We have studied the relative abundance of 28 differentially expressed proteins of resistant and susceptible variety of peanut in comparison with the respective control.

**Table 2:** Identification of differentially up-regulated proteins by MALDI-TOF-TOF in resistant and susceptible peanut upon interaction with *Phaeoisariopsis personata*.

Spot No.	Protein identified	Accession no.	Peptide sequence matched	TheoreticalMr/pI	Observed Mr/pI	S.C. (%)	MS/MS Score	Related function
Ad-1	Ribulose-bisphosphate carboxylase activase common tobacco [fragment]	gi|100380	IVDTFPGQSIDFFGALR	26.0/5.01	27/5.9	7	83	Photosynthesis
Ad-2	Oxygen-evolving enhancer protein 2, chloroplastic [*Brassica juncea*]	gi|131390	SITDYGSPEEFLSQVNYLLGK	23.4/4.91	24/5.7	9	79	Regulation of photosystem-II
Ad-3	Chloroplast ribulose-1,5-bisphosphate carboxylase/oxygenase [*Morus alba*]	gi|119855475	VPIIVTGNDFSTLYAPLIR	27.3/4.76	16.5/4.8	11	90	Photosynthesis
			IVDTFPGQSIDFFGALR					
Ad-4	Defensin like protein [*Solanum tuberosum*]	DF322_SOLTU	FSGGNCHGFRR	8.8/9.33	9/6.8	33	58	Defence
			MGPMRIAEAR					
Ad-5	Terpenoid synthase [*Arabidopsis thaliana*]	TPS08_ARATH	DPQESNR	69.5/6.15	29/6.7	3	68	Secondary metabolite biosynthesis
			FPPSEWTNR					
Ad-6	Ribulose bisphosphate carboxylase/oxygenase activase, chloroplastic [*Hordeum vulgare*]	RCAB_HORVU	VPIIVTGNDFSTLYAPLIR	47.4/7.59	43/5.4	8	127	Photosynthesis
			LVDTFPGQSIDFFGALR					
Ad-7	Sedoheptulose-1,7-bisphosphatase, chloroplastic [*Arabidopsis thaliana*]	S17P_ARATH	LLFEALQYSHVCK	42.7/6.17	34/5.1	8	170	Metabolism
			GFPGTHEFLLLDEGKWQHVK					
Ad-8	Ribulose bisphosphate carboxylase/oxygenase activase 2, chloroplastic [*Nicotiana tabacum*]	RCA2_TOBAC	VPIIVTGNDFSTLYAPLIR	48.5/8.14	49/5.2	8	181	Photosynthesis
			IVDTFPGQSIDFFGALR					
Ad-9	Putative F-box protein [*Arabidopsis thaliana*]	FB217_ARATH	LCLMACVKARDMR	46.2/8.44	45/5.9	4	68	Signal transduction & regulation of cell cycle
			NQSKEDESR					
Ad-10	Phytochrome A [*Aristolochia tomentosa*]	gi|75674163	MICDCYAKPVKVYQDER	27/6.33	28/5.4	16	80	Signal transduction
			TQTLLCDMLLRDSPLSIVSR					
Ad-11	Glyoxalase I [*Picea sitchensis*]	gi|116781841	ITSFLDPDGWK	32.8/5.04	33/5.6	3	66	Metabolism
Ad-12	Dihydroflavonol reductase [*Medicago truncatula*]	gi|357458089	ETGFDVVMINPGTALGPLIPPR	35.2/5.63	34/5.8	9	56	Secondary metabolism
			HLCVEAIR					
Ad-13	Glyceraldehyde-3-phosphate dehydrogenase [*Populus trichocarpa*]	gi|224061855	VVAWYDNEWGYSQR	47.5/6.79	47/6.6	6	101	Metabolism/Defence
			GVLDVCDVPLVSVDFR					
Ad-14	Malate dehydrogenase, cytoplasmic [*Beta vulgaris*]	gi|11133601	ELVADDAWLNGEFITTVQQR	35.8/5.89	37/6.3	6	60	Metabolism
Ad-15	Monodehydroascorbate reductase like isoform 1 [*Glycine max*]	gi|50400859	AAEEGKTVEEYDYLPYFYSR	46.9/5.73	47/5.8	4	72	Defence
Ad-16	Photosystem II stability/assembly factor [*Medicago truncatula*]	gi|357473927	FIDDKKGFVLGNDGVLLR	43.7/7.74	43/5.4	4	64	Photosynthesis
Ah-1	Chlorophyll a/b-binding protein type III, partial, [*Alonsoa meridionalis*]	gi|7271947	WLAYGEIINGR	20.8/5.17	22/5.80	19	195	Photosynthesis
			GLGGSGDPAYPGGPFFNPLGFGKDEK					
Ah-2	Light-harvesting chlorophyll a/b-binding protein [*Prunus persica*]	gi|556367	NRELEVIHSR	28.3/5.3	27.5/4.9	8	83	Photosynthesis
			NVSSGSPWYGPDR					
Ah-3	Ferritin-3, chloroplastic [*Vigna unguiculata*]	FRI3_VIGUN	IAEYVTQLR	28.5/5.54	28/5.4	9	90	Iron homeostasis, ferroxidase activity
			FFKESSEEEREHAEK					
Ah-4	Photosystem II stability/assembly factor [*Arabidopsis thaliana*]	gi|15237225	GFGILDVGYR	44.1/6.79	43/5.4	10	244	Photosynthesis
			GTGITEEFEEVPVQSR					
			SAEMVTDEGAIYVTSNR					
Ah-5	Ribulose bisphosphate carboxylase, small chain [*Phaseolus vulgaris*]	gi|21050	EVDYLLR	20.3/9.16	16/5.8	8	82	Photosynthesis
			IIGFDNVR					
Ah-6.	Ferredoxin-NADP reductase, isozyme, [*Nicotiana tabacum*]	FENR1_TOBAC	ITGDDAPGETWHMVFSTEGEVPYR	40.7/8.37	38/6.2	11	71	Regulating cyclic and non-cyclic electron flow
			DPNATVIMLATGTGIAPFR					
Ah-7	Ribulose bisphosphate carboxylase activase [*Nicotiana tabacum*]	gi|100380	VPIIVTGNDFSTLYAPLIR	26/5.01	40/5.8	22	195	Photosynthesis
			IVDTFPGQSIDFFGALR					
			LLEYGNMLVQEQENVKR					
Ah-8	Phosphoglycerate kinase, cytosolic [*Triticum aestivum*]	gi|129916	KLASVADLYVNDAFGTAHR	42.1/5.64	47/6.1	4	118	Energy Metabolism
Ah-9	RUBISCO activase, [*Cucumis sativus*]	gi|266893	VPIIVTGNDFSTLYAPLIR	45.9/7.57	40/5.9	8	224	Photosynthesis
			LVDTFPGQSIDFFGALR					
Ah-10	Cytosolic ascorbate peroxidase [*Vigna unguiculata*]	gi|1420938	YAADEDAFFADYAAAHQK	27/5.64	27/5.9	7	72	Cellular antioxidant
Ah-11	Phosphoglycerate kinase, chloroplastic [*Chlamydomonas reinhardtii*]	gi|1172455	KLAANADLYVNDAFGTAHR	49.2/8.84	48/6.4	4	188	Energy Metabolism
			LAANADLYVNDAFGTAHR					
Ah-12	Alanine aminotransferase 2 [*Glycine max*]	gi|351724369	IIFTNVGNPHALGQKPLSFPR	53.8/5.42	54/6.3	12	157	Metabolism
			MVIINPGNPTGQCLSEANLR					
			NVVCNFTEGAMYSFPQIR					

Ad- *Arachis diogoi* (Resistant), Ah- *Arachis hypogaea* L. (Susceptible)

### Identification and analysis of differentially regulated proteins

**Fig 5:**
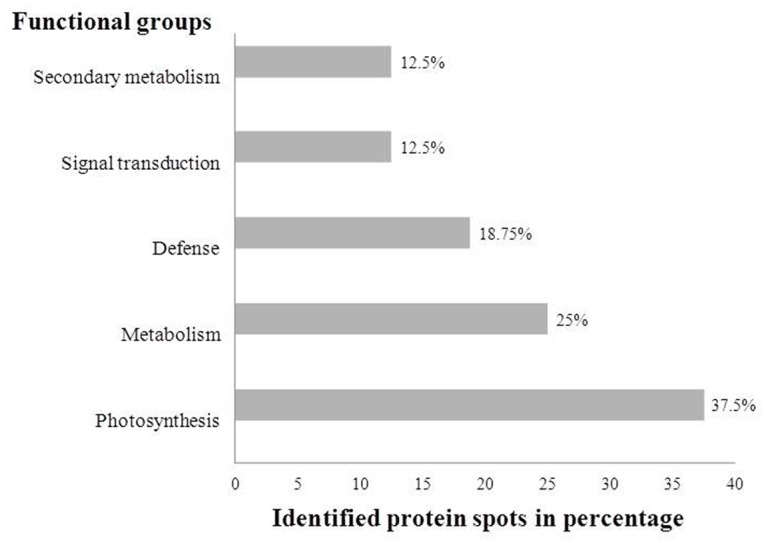
Classification of differentially expressed proteins identified. A total of 16 proteins of *Arachis diogoi* were classified based on homology search in database and their functional role in plant.

According to Bevan’s [50] method, the identified proteins obtained from *Arachis diogoi* (resistant) were grouped into functional categories based on their homology to known proteins. A major group of 6 spots (37.5%) coincided with photosynthesis related proteins, while 4 spots (25%) were designated as metabolism proteins. Proteins involved in signal transduction and secondary metabolism were found to be share an equal number of 2 (12.5%) spots. The proteins associated to defence responses were found 3 (18.75%) spots (Fig. 5). The differentially expressed proteins from resistant and susceptible peanut plants were listed in Table 2. Differentially expressed proteins related to photosynthesis are Oxygen-evolving enhancer protein, Ribulose-bisphosphate carboxylase activase, Light-harvesting chlorophyll a/b-binding protein and Photosystem II stability/assembly factor. Metabolism related proteins are Sedoheptulose-1,7-bisphosphatase, Glyoxalase I and Malate dehydrogenase. Proteins involved in secondary metabolism are Dihydroflavonol reductase and Terpenoid synthase. Protein identified related to signal transduction and defence are Putative F-box protein, Phytochrome A and Defensin-like protein, Monodehydroascorbate reductase, Glyceraldehyde-3-phosphate dehydrogenase respectively.

We also observed several differentially regulated protein spots in the *Arachis hypogaea* cv. JL-24 (Susceptible) upon pathogen inoculation. We have successfully identified 12 proteins, while the rest showed no significant hits in the database. We found that proteins involved in energy metabolism, such as Phosphoglycerate kinase and Ferredoxin-NADP reductase were up-regulated along with other metabolism related protein like alanine aminotransferase. Moreover, most of the identified leaf proteins were found to be related to photosynthesis. Three up-regulated spots showed similarity to Rubisco activase and two was similar to Chlorophyll a/b binding protein, while another spot was identified as cytosolic ascorbate peroxidase with antioxidant properties. Identified proteins showed involvement in stress tolerance, energy metabolism, photosynthesis and protein synthesis under stress conditions. The details of the proteins identified using proteomic approach were presented in Table 2. The predicted molecular weight (MW) and *p*I of all these proteins were compared with their positions on the gel and were found consistent with some exception, which might be due to post-translational modifications or due to polymeric nature of the proteins. The matched peptide sequence and its number, reference organism, sequence coverage, experimental and theoretical molecular weight and *p*I, MS/MS score and related function of each protein were determined (Table 2). RuBisCO was identified as the most abundant and differentially expressed protein, which could be due to presence of different isoforms of same protein or could be due to post-translational modification.

### qRT-PCR analysis of different TDFs to validate cDNA-AFLP results

**Fig 6:**
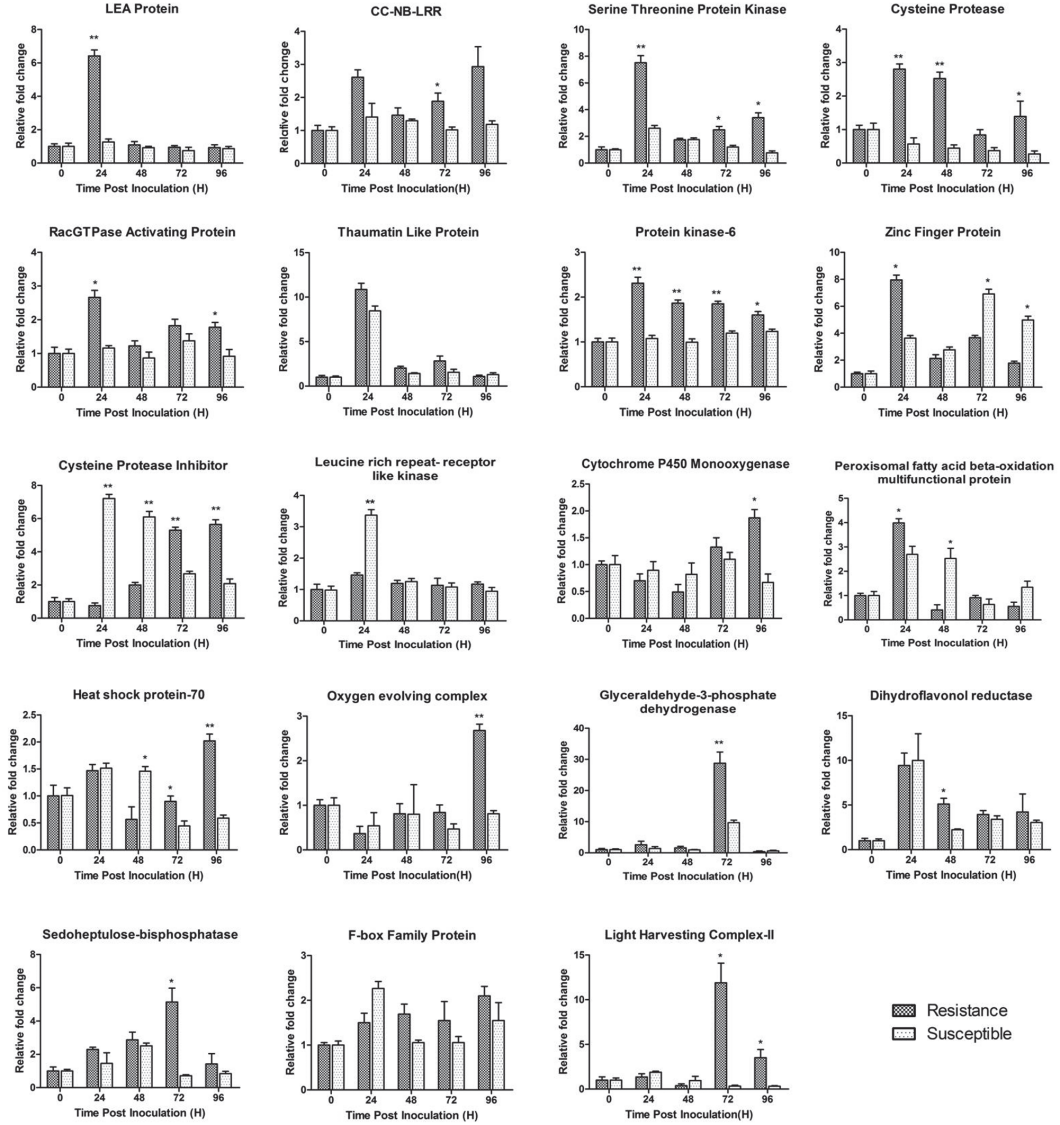
Quantitative real-time PCR (qRT-PCR) analyses of 19 selected TDFs. Leaf tissues were used for both inoculated and mock inoculated plants at 24, 48, 72 and 96 hpi, as well as mock-inoculated near 0 hpi. Relative gene quantification was calculated by comparative ΔΔCT method. All data were normalized to the Alcohol dehydrogenase-3 and 60S ribosomal protein expression level as these were used as internal reference gene and data from three biological replicates mean ± SD was plotted. Statistical analysis was performed with Student’s t-test, asterisks indicate a significant difference between resistant (*Arachis diogoi)* and susceptible (*Arachis hypogaea* cv. JL-24) (* P < 0.05, ** P < 0.01).

Nineteen differentially expressed TDFs, of which most of them were related to defence, signal transduction and metabolism, were selected for validating the results obtained from cDNA-AFLP analyses using qRT-PCR. In this analysis, a comparison has been made for the expression of the chosen genes between the resistant wild peanut and the susceptible cultivated peanut at similar time points (0, 24, 48, 72 and 96 hpi) after the pathogen challenge and illustrated in Fig. 6 and S2 Table. This study showed that most of the gene fragments related to signal transduction and defence such as CC-NB-LRR, racGTPase activating protein, serine threonine protein kinase, zinc finger protein, thaumatin like protein (*TLP*), Protein kinase-6, late embryogenesis abundant (*LEA*) protein and cysteine protease were found to be upregulated within 24 h after the pathogen treatment in the resistant genotype compared to the susceptible cultivated genotype, which showed no such upregulation. Cysteine protease inhibitor has shown early response in the susceptible peanut plants i.e, upregulated at 24 and 48 h, while the wild peanut plants showed late response at 72 and 96 hrs. Leucine rich repeat- receptor like kinase (LRR-RLK) was found to be up-regulated in susceptible plants at 24 h, while no change was observed in the resistant plants, which was in agreement with our cDNA-AFLP data, where it was found to be downregulated. It is possible that it might be involved in downstream signaling in relation to pathogen recognition. Cysteine protease showed contrasting expression patterns. While it was observed downregulated upon pathogen challenge in cDNA-AFLP analysis, but was found to be upregulated upon validation by qRT-PCR analysis. Similarly, a zinc finger protein was found to be down-regulated in cDNA-AFLP analysis as against a second transcript that has shown up-regulation.

We examined transcript levels of six selected differentially expressed gene through quantitative real-time PCR, which were also found to be differentially expressed at 2-D proteomic analysis. We have found glyceraldehyde-3-phosphate dehydrogenase to be up-regulated within 24 hrs after the pathogen treatment in the resistant genotype compared to the susceptible cultivated genotype, which showed no such up-regulation. Photosystem II regulatory protein and oxygen evolving enhancer complex was found to be up-regulated at 72 and 96 hrs in the wild peanut respectively, while there was no such up-regulation in the susceptible peanut plants. Sedoheptulose-bisphoshatase, a metabolism related protein was found up-accumulated constantly up to 72 hrs in wild peanut compared to susceptible peanut, which did not evidence its upregulation. A secondary metabolism protein, dihydroflavonol reductase has shown early response in the wild as well as susceptible peanut plants i.e, upregulated at 24 hrs, and gradually decline at 48, 72 and 96 hrs. F-box family proteins that regulate diverse cellular processes was found to be up-regulated at different time point in both resistant as well as susceptible peanut.

Of the total nineteen gene fragments, gene expression patterns of seventeen were similar to those observed in cDNA-AFLP and 2-D gel electrophoresis analysis except cysteine protease and zinc finger protein which were found downregulated in cDNA-AFLP analysis. The results indicate that cDNA-AFLP data and 2-D protein expression profile are mostly concordant with qRT—PCR data, confirming the reliability of the results with minimum discrepancies. The transcripts of a LEA protein, cysteine protease, serine-threonine protein kinase, glyceraldehyde-3-phosphate dehydrogenase, oxygen evolving complex, sedoheptulose-bisphoshatase and Photosystem II regulatory protein showed very clear differences between the resistant and susceptible genotypes with the wild peanut showing strong upregulation.

## Discussion

Peanut is the most important oilseed-protein crop of semi-arid tropics with equal importance word-wide. This crop is highly susceptible to various foliar diseases, particularly the late leaf spot disease; and the damage to the crop can reach up to 70% under severe epidemic conditions, particularly when the crop receives an extended rainy season. Molecular studies on the peanut foliar diseases are very rare and the crop did not receive due attention it deserves. There were only two reports on the characterization of genes involved in peanut (*A*. *hypogaea*) -*P*. *personata* interaction [16,52] and also two reports in the wild relatives of *Arachis* that are highly resistant to several pathogens including *P*. *personata* [19,53]. Kumar and Kirti [19] reported several transcripts associated with phenylpropanoid pathway, such as phenylalanine ammonia-lyase, cinnamate 4-hydroxylase, cinnamyl alcohol dehydrogenase with significant roles in first line of defence such as cell wall deposition and lignifications. They have also reported the upregulation of a pathogen induced cyclophilin and characterized it and showed that constitutive expression of cyclophilin in tobacco has been associated with enhanced resistance against pathogen *R*. *solanacearum* and *P*. *parasitica* var. *nicotianae* [19].

Since the peanut genome sequence is not yet available in the international GenBanks, it is important to discover novel genes through alternative transcriptomic approaches. cDNA-AFLP method is a powerful approach for studying the differential gene expression and we used this technique to provide first large scale investigation of the genes expressed in the resistant wild peanut in its incompatible interaction between *Phaeoisariopsis personata*. This technique has been used successfully to study plant-pathogen interaction in several plants [26,54,55] and the results are reported to be highly reproducible [56] and have advantages over other commonly used gene display methods [57].

In the present study, the 233 differentially expressed gene fragments were grouped into functional categories based on their homology to known proteins. A major group of sequences showed no significant similarity while most of the sequences were designated unknown/hypothetical protein. The genes showing significant similarity to metabolism, photosynthesis, signal transduction, defence, transport and transcription factors were illustrated in Table 1. Nineteen differentially expressed TDFs were further validated by quantitative real time PCR. Of these, nine TDFs exhibited early response as they were up-regulated at 24 h time point after pathogen inoculation in resistance plants, while there was no major upregulation in susceptible plants except thaumatin like protein. Thaumatin like protein showed an early response as it was upregulated at 24 h after infection in compatible as well as incompatible interaction. This 24 h point also includes the time taken for the conidia to germinate. Nine TDFs exhibited late response in resistant plants and their transcripts were up-regulated at 72 and 96 h, while trivial changes were found in susceptible plants except cystatin. Cysteine protease inhibitor has shown early response as it is up-regulated at 24 and 48 h after infection in compatible interaction.

We have detected four transcripts with similarity to thaumatin like protein, a PR5 protein, which exhibited antifungal activity in vitro [58,59] as well as in vivo [60,61]. Cotton thaumatin like protein (*GbTLP*) in tobacco transgenic plants enhanced resistance against *Verticillium dahliae* [62]. It showed differential expression profile in wild peanut in the cDNA-AFLP analysis, but there was no significant difference between compatible and incompatible interaction in quantitative real time analysis. Another transcript similar to cystatin has been found to be up-regulated both in cDNA-AFLP as well as quantitative real time analysis, and exhibit antifungal properties in several plants [63,64]. We have observed three transcripts of a disease resistance protein similar to CC-NB-LRR protein in our analysis. It is an R gene that confers resistance to various plant fungal pathogens [65,66] and has also been shown to impart leaf stripe resistance in barley [67]. Quantitative real time analysis also indicated up regulation in wild peanut, which could not be observed in susceptible peanut at various time points of pathogen inoculation. Two TDFs of 15-Hydroxyprostaglandin dehydrogenase, a homolog of NADPH oxidoreductase have been found in cDNA-AFLP analysis, which are reported to be involved in generating reactive oxygen species such as O_2_ˉ & H_2_O_2_ for exhibiting hypersensitive response [68,69]. It has also been shown to be involved in the suppresssion of human breast cancer and also can modulate estrogen receptor pathway [70]. *Arabidopsis thaliana* 15-Hydroxyprostaglandin dehydrogenase plays a distinct role in plant anti-oxidant defence [71]. This protein might be playing an important role in preventing pathogen invasion.

Protein kinases are known to play important roles in pathogen recognition through signaling and activation of plant defence mechanisms [72] through the phosphorylation of the target proteins. We have identified several TDFs encoding different protein kinases such as Leucine rich repeat receptor like kinase (LRR-RLK), Protein kinase-6, Serine-threonine protein kinase [16], Flag-tagged protein kinase domain of putative mitogen-activated protein kinase kinase kinase. Except the LRR-RLK, all other protein kinases were found to be induced in the wild peanut, while LRR-RLK was repressed in both cDNA-AFLP as well as quantitative real time analysis. Sugarcane LRR-RLKs have been shown to have roles in downstream signalling pathway, particularly in relation to endophytic bacterial association [73].

We have identified transcripts of proteins like vacuolar processing enzyme and cysteine proteases that are associated with cell death and induced during pathogen infection with in hypersensitive response [74]. Vacuolar processing enzyme has been reported as a cysteine proteinase that is responsible for the maturation of vacuolar proteins and exhibits caspase-1 like activity [75,76]. It exhibits endopeptidase activity and mediates TMV and mycotoxin-induced cell death [77,78]. We have found a cysteine protease, another cell death associated gene fragment in our studies and it has been shown to be involved in senescence [79] and various environmental stresses including hypersensitive cell death [80].

SGT1 is an essential component of signaling pathways leading to pathogen resistance and binds to the chaperone proteins, HSP70 in plants [81] and HSP90 in human, yeast, plants [82,83] indicating its role in regulating protein folding. SGT1 regulates defence responses triggered by various pathogens and interacts with RAR1 [84] and is essential for resistance conferred by multiple *R* genes [85,86]. It plays an important role in regulating process of cell death during compatible and incompatible plant-pathogen interaction [27].

In the present study, racGTPase was found to be differentially expressed and in quantitative RT-PCR analyses shown its expression reached the peak at 24 hpi in the resistant genotype, while no major changes in its expression were observed in susceptible variety. Rac GTPase protein plays an important role in plant defence against pathogen and its role in the production of reactive oxygen species (ROS) such as O_2_ˉ and H_2_O_2_ that are rapidly generated after infection leading to hypersensitive response, a form of programmed cell death in plants has been very clearly elucidated [87]. The expression of a rice racGTPase resulted in HR like response and resistance against a virulent strain of bacterial blight and blast fungus associated with an altered expression of defence related genes involved in the enhanced production of phytoalexins [88]. However, its function in R gene mediated disease resistance still needs to be established.

Several metabolism related proteins were found to be differentially expressed such as sedoheptulose biphosphatase (SBPase), LEA protein, methionine synthase, cellulose synthase, Exostosin like protein, glycine rich protein, NADH dehydrogenase, glyceraldehyde phosphate dehydrogenase, peroxisomal fatty acid β-oxidation multifunctional protein, dihydroflavonol reductase, UDP glucosyl transferases, GDSL-like Lipase/Acylhydrolase. ABC transporters etc. in the resistant genotype in comparison to the susceptible variety.

We have quantitatively estimated the expression of LEA protein, sedoheptulose-1,7- biphosphatase (SBPase), Photosystem II type I chlorophyll a/b-binding protein and peroxisomal fatty acid beta- oxidation and observed that LEA protein and peroxisomal fatty acid β-oxidation multifunctional protein were at peak at 24 hrs in resistant genotype, while there was no such upregulation in the susceptile variety. SBPase and Photosystem II type I chlorophyll a/b-binding protein trsnscript accumulation was strong and constantaly increased upto 72 hpi in the resistant genotype compared to the susceptible one. SBP is a calvin cycle enzyme and stimulates photosynthesis and growth from an early stage of development has been reported in transgenic tobacco upon overexpression [89]. SBPase was found to be differentially expressed in *Arabidopsis thaliana* upon infection with tobacco etch virus [90]. Hence, SBPase might have significant role in the resistance phenomenon in the wild peanut and needs to be further investigated.

Most of these ESTs were not identified in previous studies of *Arachis hypogaea* and *Phaeoisariopsis personata* interaction at molecular level using subtractive suppression hybridization [16], as well as ESTs libraries using microarray technique [52]. This clearly shows the sensitivity and importance of cDNA-AFLP in identifying novel genes induced in different stresses. Kumar and Kirti [19] reported up regulation of phenylpropanoid pathway genes such as phenylalanine ammonia lyase, cinnamate 4-hydroxylase, cinnamyl alcohol dehydrogenase and dirigent-like protein upon interaction between wild peanut and *P*. *personata*. Previous reports showed the upregulation of serine threonine protein kinase, heat shock protein, ABC transporter protein in *Phaeoisariopsis personata* and *Arachis hypogaea* interaction [16]. Similarly, Kumar and Kirti [19] reported on the expression of genes such as zinc finger protein, thaumatin like protein, methionine synthase, ATPase, nucleic acid binding protein, heat shock proteins, cysteine protease, oxygen evolving enhancer protein and a receptor kinase in the wild peanut, *Arachis diogoi* challenged with *P*. *personata* using the approach of DD-RT-PCR. It is interesting to note that similar genes have been identified using the cDNA-AFLP approach in the present study showing the importance of these genes in the resistance phenomenon. Hence disease resistance associated genes such as CC-NB-LRR, SGT1, cystatin, protein kinases, racGTPase activating protein, Cytochrome P450 monoxygenase, vacuolar processing enzyme, heat shock 70 kDa protein and 15-Hydroxyprostaglandin dehydrogenase, which were not reported in the previous studies could serve as novel candidate resistance genes in the development of disease resistant variety of peanut.

In order to study the proteins showing differential expression in the resistant and susceptible genotypes of peanut with response to late leaf spot pathogen, *Phaeoisariopsis personata*, we have followed a proteomic approach. We have successfully identified 16 protein spots with significant expression in wild peanut and 12 protein spots in susceptible peanut, because the importance of the proteins involve in incompatible interaction from wild peanut. Identified proteins could be divided into functional groups including photosynthesis, metabolism, secondary metabolism, signal transduction and defence related proteins (Fig. 6).

RuBisCO activase is the most abundant and important photosynthetic enzyme in C_3_ plants which leads to carbon fixation and photorespiration. The RuBisCO enzyme found in the chloroplast plays an important role in photosynthesis and is known to be reduced in infected plant cells because attack of pathogens lead to degradation of chloroplasts [91]. The upregulation of large subunit of rubisco (rbcL) and other photosynthesis related proteins has been reported in rice plants under stress [92,93] and a similar phenomenon has been observed both in susceptible/resistant genotypes during plant-pathogen interaction in the present study. Wu, et al. [38] reported that Rubisco was differentially expressed in resistance and susceptible genotypes of maize infected with sugarcane mosaic virus. Several ribulose bisphosphate corboxylase enzymes were found up-accumulated in wild peanut, while it was also differentially up-regulated in susceptible peanut. Photosynthesis is carried out by two light dependent part, photosystem I and photosystem II, in thylakoid membrane of chloroplasts. The chlorophyll a/b binding protein stabilizes the photosystem I and II through balanced excitation energy. In our study, we have observed an increased expression of chlorophyll a/b binding protein in susceptible peanut, which is similar to Wu, et al. [38] finding, where it was induced in susceptible maize upon virus inoculation. We have also found differential expression of chlorophyll a/b binding protein upon pathogen challenge in wild peanut. We have identified induced expression of photosystem II stability factor in both wild and susceptible peanut, indicating its possible role in disease resistance. According to Metha, et al. [94], photosystem II has emerged as a target of resistance signalling in plant-pathogen interaction. Oxygen evolving enhancer protein is another photosystem II associated protein, which is involved in the regulation of photosystem II and was differentially up-regulated upon pathogen challenge in wild peanut in proteomic study and also in cDNA-AFLP analysis. Quantitative real-time analyses validate the up-regulation of oxygen evolving enhancer protein in wild peanut, while no such up-regulation was found in the susceptible peanut plants. Recently, differentially expressed genes were identified in resistant maize genotypes upon virus infection [38]. The results indicate that the ability of photosynthesis of resistance and susceptible peanut genotype might be different due to chloroplasts of the sampled cells were affected by the infection, and photosynthetic activity was increased possibly to compensate the loss.

F-box proteins regulate diverse cellular processes, including cell cycle transition, transcriptional regulation and signal transduction by playing roles in Skp1p-cullin-F box protein (SCF) complexes. F-box proteins have also been reported to be expressed during panicle and seed developmental stage and therefore appear to be involved in regulating plant growth and development. Wang, et al. [95] have demonstrated the role of F-box protein during various stress responses like water deficit, salts, wounding, and elicitation. In our study, we have found F-box protein differentially up-regulated in wild peanut upon pathogen inoculation and quantitatively validated its up-regulation by real-time analyses in resistant as well as susceptible peanut plants.

Glyceraldehyde-3-phosphate dehydrogenase (GAPDH) is a central glycolytic protein with pivotal role in energy production. Recent studies, in animal system indicating a role of glyceraldehyde-3-phosphate dehydrogenase (GAPDH) in apoptosis or oxidative stress has been reported [96,97]. GAPDH is also involved in various diseases especially neurodegenerative disorders and cancers [98]. We have found differential up-regulation of GAPDH in wild peanut upon pathogen challenge in proteomic study as well as cDNA-AFLP analysis. Quantitative real-time analyses showed strong up-regulation of GAPDH at 72 hrs of pathogen inoculation in wild peanut, while trivial up-regulation was observed in the susceptible peanut plants. Recently, GAPDH was found up-regulated in a proteomic study during plant-virus interaction in maize genotypes with sugarcane mosaic virus [38].

In plants, ascorbic acid plays an important role in stress responses as well as growth and development and act as an antioxidant to scavenge reactive oxygen species (ROS) generated during physiological processes [99]. Ascorbic acid is also involved in defence mechanisms against pathogen attack and environmental oxidative stresses, and has been implicated in the regulation of cell division and expansion [100]. Ascorbic acid is readily oxidized to monodehydroascorbate, which dissociates to form unstable dehydroascorbate at alkaline pH. Monodehydroascorbate reductase (MDAR) and dehydroascorbate reductase (DHAR) are known to play protective roles in plants as enzymes that maintain ascorbate in its reduced form [101,102]. Monodehydroascorbate reductase uses NAD(P)H as a reductant and maintains reduced pools of ascorbate by recycling the oxidized form of ascorbate, which serves as an important antioxidant [103]. Yoon, et al. [104] demonstrated increased mRNA expression of *Brassica campestris* monodehydroascorbate reductase in response to oxidative stress invoked by hydrogen peroxide, salicylic acid, paraquat, and ozone. In our study, monodehydroascorbate reductase was induced upon pathogen challenge in highly resistant wild peanut genotype. Therefore, we can assume that MDAR might be involved in controlling the balance amount of antioxidant in the cell during pathogen challenge, which has to be further validated for better understanding.

Plant defensins are a family of small, basic proteins that contain 4–5 disulfide bonds and known to possess potent antifungal activity. The majority of characterized plant defensins show a constitutive pattern of expression, with an induction in expression in response to pathogen attack, wounding and some abiotic stresses [105,106]. Plant defensins are best known for their antimicrobial activity against a broad spectrum of plant pathogens that include bacteria, yeast and a number of pathogenic fungi [107]. We observed induced expression of a defensin protein in wild peanut upon pathogen challenge while unable to detect in susceptible peanut. It could be one of the important reasons for resistivity of wild peanut against micro-organisms.

Sedoheptulose-1,7-biphosphatase (SBP) was found up-accumulated in the resistant genotype of peanut. SBPase is a Calvin cycle enzyme and stimulates photosynthesis and growth from an early stage of development in transgenic tobacco upon overexpression [89]. It was found to be differentially expressed in *Arabidopsis thaliana* upon infection with tobacco etch virus [90]. We have also found that proteins involved in secondary metabolism, such as Terpenoid synthase and Dihydroflavonol reductase were up-regulated along with other metabolism related proteins like glyoxalase I and malate dehydrogenase. We have observed proteins that are involved in signal transduction such as phytochrome-A and F- box protein were found differentially up-regulated in wild peanut upon pathogen challenge. Hence, these proteins through metabolism and siganaling, might have significant role in the defence phenomenon in the wild peanut and needs to be further investigated.

We found several photosynthesis related proteins like Rubisco activase, Chlorophyll a/b binding protein and Ferredoxin-NADP reductase up-regulated in the susceptible peanut upon pathogen challenge. Rubisco is the abundant enzyme of plant cell and plays important role in photosynthetic carbon fixation. Wu, et al. [38] identified its up-regulation during plant-virus interaction in resistant and susceptible ecotypes of maize infected with sugarcane mosaic virus. Chlorophyll a/b binding protein provides excitation energy between photosystem I and II, which balances the photosystems [108]. Chlorophyll a/b binding protein upregulation was also reported in *Brassica juncea* and maize during plant-pathogen interaction [37,38]. Ferredoxin-NADP reductase transfers electrons between the electron carriers, ferredoxin and NADP(H) in the photosynthetic electron transport system and up-regulated upon biotic stress [38]. Phosphoglycerate kinase catalyzes the reaction of 1,3-Biphosphoglycerate and ADP to produce 3-Phosphoglycerate and ATP. This method for ATP production is known as substrate-level phosphorylation because it produces energy storing ATP molecules without the use of oxygen, NADH, or an ATPase. We have found up-regulation of two spots corresponding to phosphoglycerate kinase. Kaur, et al. [37] also observed the up-regulation of phosphoglycerate kinase in proteome analysis of *Albugo candida- Brassica juncea* pathosystem. Cytosolic ascorbate peroxidase possesses anti-oxidative properties and protects cellular components such as mitochondria and chloroplasts against oxidative stress. Similarly, Wu, et al. [38] observed its up-regulation during plant-virus interaction in maize with sugarcane mosaic virus. Identified proteins were involved in stress tolerance, energy metabolism, photosynthesis and protein synthesis under stress conditions.

Taken together cDNA-AFLP as well as 2D analysis data, we observed that the oxygen-evolving enhancer protein, F-box protein, Sedoheptulose-1,7-biphosphatase, Glyceraldehyde-3-phosphate dehydrogenase, Dihydroflavonol reductase, Phytochrome A and Photosystem-II chlorophyll a/b binding protein were up-regulated upon pathogen challenge. This is the first approach to elucidate the molecular basis of the response of the resistant genotype to the late leaf spot pathogen, and its defence mechanism. How plants regulate the photosynthetic apparatus and increases systemic pathogen resistance during defence however, remains unclear and further studies are required. Hence, these TDFs and proteins might have significant role in the resistance phenomenon in the wild peanut and need to be further characterize. Our results anticipate the cloning resistance genes for tikka disease from wild peanut *Arachis diogoi* that would facilitate generation of pathogen-resistant peanut cultivars.

## Supporting Information

S1 Fig *Phaeoisariopsis personata* infection and spore germination, (a) Spore infection in *Arachis hypogaea* (Susceptible) 24 dpi and spores, (b) Spore infection in *Arachis diogoi* (Resistant) 24 dpi and germinated spore 24 hrs. (TIF)

S2 Fig cDNA-AFLP gel picture: A representative picture of cDNA-AFLP gel showing differential expression of TDFs upon pathogen challenge in *Arachis diogoi*. C- represents the pool sample of mock inoculated at 24,48,72 and 96 hrs while T- represents the pool sample of pathogen inoculated at 24,48,72 and 96 hrs. The primer combination used were; lane C_1_T_1_: M-CAG/E-AGG, C_2_T_2_: M-CAG/E-ACG, C_3_T_3_: M-CAG/E-AGC, C_4_T_4_: M-CAG/E-ACC. Arrow indicate differentially expressed TDFs selected for further analysis.
(TIF)

S1 Table Primers used in quantitative real time PCR analysis. (DOCX)

S2 Table Quantitative validation of nineteen TDFs, which were differentially expressed in incompatible (Resistance = *A*. *diogoi*) interaction and compared with compatible (Susceptible = *A*. *hypogaea* L.) interaction upon *Phaeoisariopsis personata* inoculation at different time points. (DOCX)

S3 Table TDFs Submitted to GenBank. (DOCX)
