# Supplementary figures and images for: Transcriptomic and Proteomic Analyses of Resistant Host Responses in Arachis diogoi Challenged with Late Leaf Spot Pathogen, Phaeoisariopsis personata
Source: PLoS One. 2015 Feb 3;10(2):e0117559. doi: 10.1371/journal.pone.0117559 (PMC4315434; doi:10.1371/journal.pone.0117559)

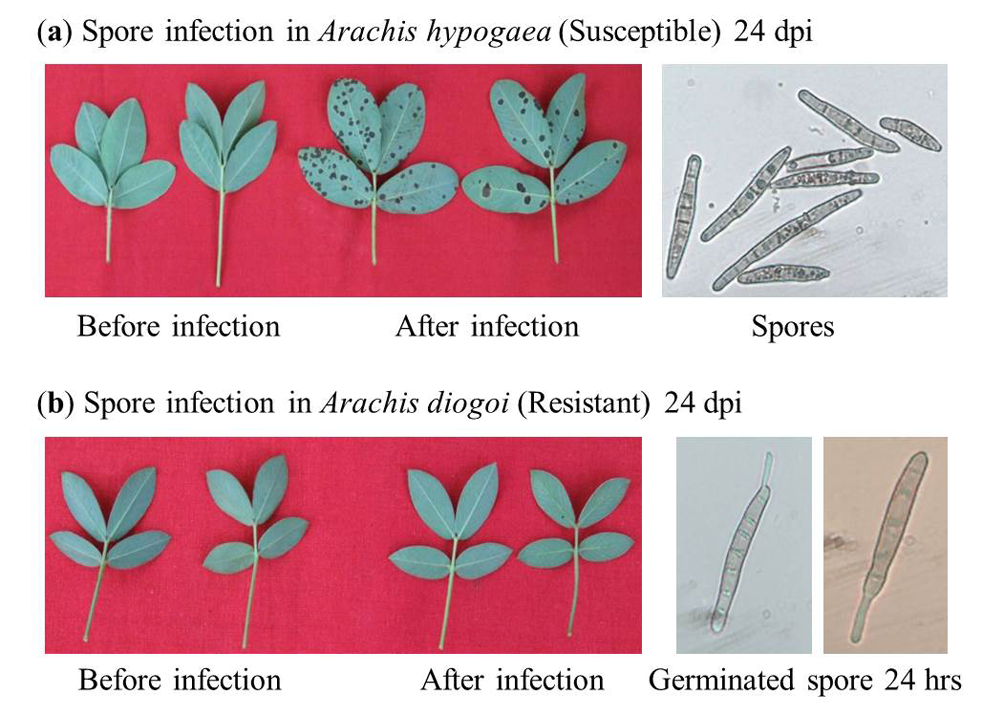

Supplement: S1 Fig — (TIF) [file pone.0117559.s001.tif]

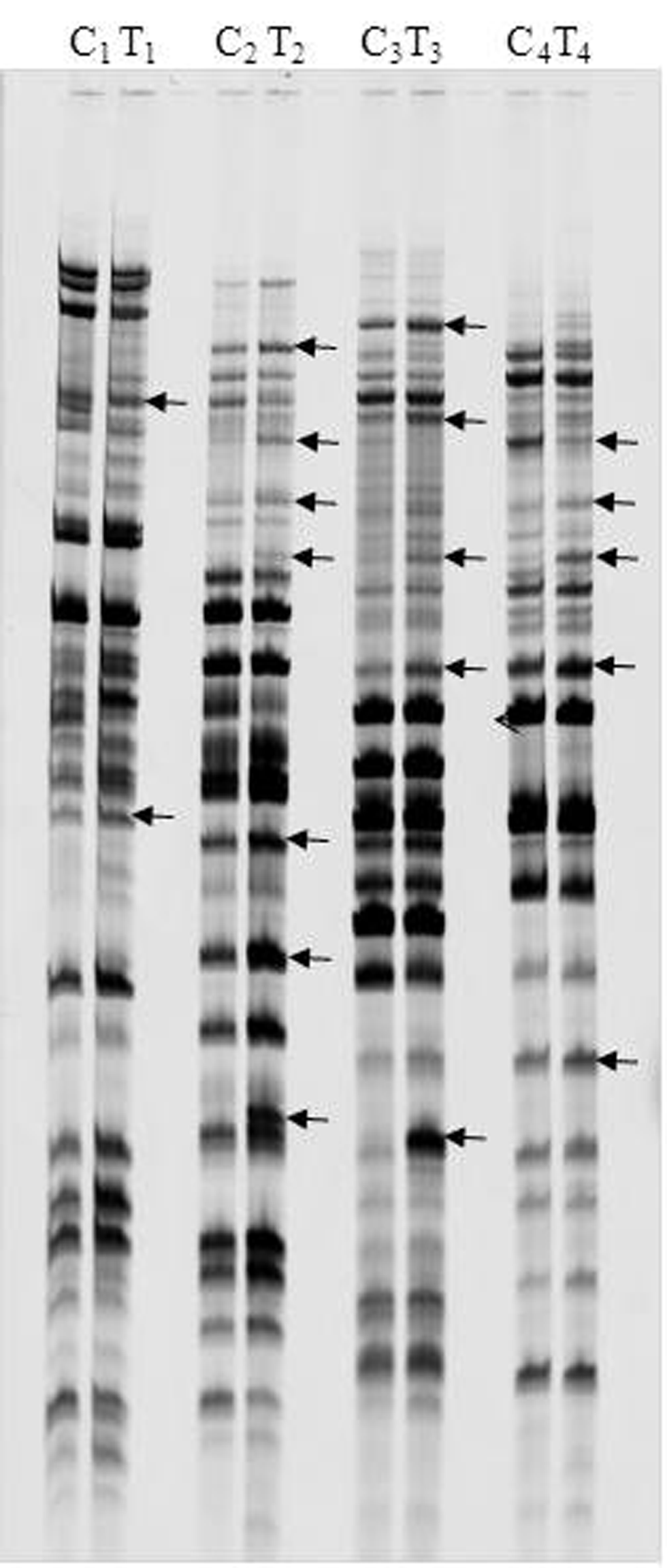

Supplement: S2 Fig — C- represents the pool sample of mock inoculated at 24,48,72 and 96 hrs while T- represents the pool sample of pathogen inoculated at 24,48,72 and 96 hrs. The primer combination used were; lane C1T1: M-CAG/E-AGG, C2T2: M-CAG/E-ACG, C3T3: M-CAG/E-AGC, C4T4: M-CAG/E-ACC. Arrow indicate differentially expressed TDFs selected for further analysis. (TIF) [file pone.0117559.s002.tif]
