# Supplementary material for: Transcriptomic and Proteomic Analyses of Resistant Host Responses in Arachis diogoi Challenged with Late Leaf Spot Pathogen, Phaeoisariopsis personata
Source: PLoS One. 2015 Feb 3;10(2):e0117559. doi: 10.1371/journal.pone.0117559 (PMC4315434; doi:10.1371/journal.pone.0117559)
Supplement: S1 Table — (DOCX) [file pone.0117559.s003.docx]

| **TDFs** | **Primer Abbreviation** | **Primer Sequence (5'-3')** | **Primer Size** | **GC in %** | **Tm in ºC** | **Amplicon Size (bp)** |
| --- | --- | --- | --- | --- | --- | --- |
| Lea Protein | AdRTLEA-F  AdRTLEA-R | GGCTTTGCATTGTGGGACATGA  TCACTCCTCGTCGTCATCGTC | 22  21 | 50.0  57.1 | 58.5  58.4 | 189 |
| Cystatin | AdRTCPI-F  AdRTCPI-R | GGAGCAAGTAGTTGCTGGAAGC  AGTGCTCTAGACACTGAAGCAGC | 22  23 | 54.5  52.2 | 58.3  58.8 | 189 |
| Zincfinger protein | AdRTZFP-F AdRTZFP-R | CTGTGTTGCCTTGTGGGCATAC  AGGTTTCTGGCATTGGAGTTGAGG | 22  24 | 54.5  50.0 | 58.9  59.4 | 162 |
| RacGTPase | AdRTRACG-F  AdRTRACG-R | CCT TGCTTCTGCTGAGTGTAAGGA  CTTCGGCCAACTCCGTTCTTG | 24  21 | 50.0  57.1 | 58.8  58.6 | 167 |
| LRR-RLK | AdRTLRR-F  AdRTLRR-R | CGGAAATGATGTCAAGTGTGGTGG  TGAAGTGTCAATTCTGCCCTCCAG | 24  24 | 50.0  50.0 | 58.3  59.1 | 164 |
| CC-NB-LRR | AdRTCNLRR-F AdRTCNLRR-R | GTGTGGAGACGTTGGAATGCAAG  CTGACATCGTTTGGTCAGCAAAGG | 23  24 | 52.2  50.0 | 58.6  58.5 | 164 |
| Cysteine protease | AdRTCP-F  AdRTCP-R | TGTCCAGGGGACGAGACATG  CCAGCATTCTGGAGGCAGAGTC | 20  22 | 60.0  59.1 | 58.9  59.8 | 170 |
| Fatty acid β-xidation | AdRTPFBO-F  AdRTPFBO-R | GCTTCAAGGAAACCCTCTGTTGC  GTCGCTGTGTTCCTCCAAAGC | 23  21 | 52.2  57.1 | 58.7  58.8 | 163 |
| HSP70 | AdRT70HSP-F  AdRT70HSP-R | GGACCAAGGCACCATGTAAGAGC  CTGCTCCCATGGCAACTGC | 23  19 | 56.5  63.2 | 60.0  59.5 | 183 |
| Serine/threonine kinase | AdRTSPK-F  AdRTSPK-R | GAAGAAGGGGAACAGATGCTGGT  GATTGGAGGATCGGCGTGTTC | 23  21 | 52.2  57.1 | 59.1  58.2 | 168 |
| Cytochrome P450 | AdRTCYP450F  AdRTCYP450R | AGCACTTGCAATGCTTGTTAGACG  ACGAGGGCACAATTGGAGGT | 24  20 | 45.8  55.0 | 58.3  59.2 | 149 |
| Protein  kinase-6 | AdRTPK6-F  AdRTPK6-R | GGACTGTGGACCTCCCTAAGC  CGAGACCAAAGCGAGACCTCTC | 21  22 | 61.9  59.1 | 59.3  59.3 | 193 |
| Photosystem II chlorophyll a/b-binding protein | AdRT-PS-II-F  AdRT-PS-II-R | TCCCTCATTGGCTGGCCAAG  ATGGGCCCAAGTACTTGACACG | 20  22 | 60.0  54.5 | 60.2  59.6 | 149 |
| Thaumatin like protein | AdRTTLP-F  AdRTTLP-R | CGTCGCGAGTTGTCCAGCTA  GTGATCGCCGGTGCAACAA | 20  19 | 60.0  57.9 | 59.5  58.9 | 157 |
| SBPase | AdRTSBP-F AdRTSBP-R | TGCAAGTATGCATGTTCCGAGGAA  CACGCCAAAGATTGTTCCAACTGT | 24  24 | 45.8  45.8 | 58.9  58.3 | 144 |
| DHFR | AdRTDHFR-F  AdRTDHFR-R | ACTTCAAGTGGCTCCGCTGTC  CACAGCCTTCTCAGCCAAAGTC | 21  22 | 57.1  54.5 | 59.8  58.3 | 147 |
| GAPDH | AdRTGAPDH-F  AdRTGAPDH-R | CAGTGGACAGAGGTGCACTGC  CCGACGATTCAGACACAACTGGAG | 21  24 | 61.9  54.2 | 60.6  59.4 | 155 |
| F-box family protein | AdRTFbox FP-F  AdRTFbox FP-R | ACGTGCATGGACGACAACTGC  GAGTGCAAGGGACCTGACCTCA | 21  22 | 57.1  59.1 | 60.6  61.0 | 120 |
| Oxygen-evolving complex | AdRT-OEChl-F  AdRT-OEChl-R | GGTGTGGCTGCCAAACTCATAG  GTCCTGTCACCGTGTAGAGTCTG | 22  23 | 54.5  56.5 | 58.4  58.6 | 157 |
| Alcohol dehy- drogenase -3 | AdRTADH3-F  AdRTADH3-R | GACGCTTGGCGAGATCAACA  AACCGGACAACCACCACATG | 20  20 | 55.0  55.0 | 57.9  58.1 | 140 |
| 60S Ribosomal protein | AdRT60SRibP-F AdRT60SRibP-R | TGGAGTGAGAGGTGCATTTG  TCTTTTGACGACCAGGGAAC | 20  20 | 50.0  50.0 | 55.0  54.9 | 155 |
