# Supplementary material for: Transcriptomic and Proteomic Analyses of Resistant Host Responses in Arachis diogoi Challenged with Late Leaf Spot Pathogen, Phaeoisariopsis personata
Source: PLoS One. 2015 Feb 3;10(2):e0117559. doi: 10.1371/journal.pone.0117559 (PMC4315434; doi:10.1371/journal.pone.0117559)
Supplement: S2 Table — (DOCX) [file pone.0117559.s004.docx]

| **Accession no.** | **Annotation** | **Blast score** | **Category** | **Incompatible interaction**  **(in hpi)** | | | | **Compatible interaction**  **(in hpi)** | | | |
| --- | --- | --- | --- | --- | --- | --- | --- | --- | --- | --- | --- |
|  |  |  |  | **24** | **48** | **72** | **96** | **24** | **48** | **72** | **96** |
| EU935215 | Cystatin [*Spinacia oleracea*] | 9e-20 | Defence | - | + | +++ | +++ | +++ | +++ | ++ | ++ |
| GQ466607 | Thaumatin-like protein 1a-like [*Glycine max*] | 7e-93 | Defence | ++++ | + | +++ | + | +++ | + | + | + |
| GU592820 | CC-NB-LRR type disease resistance protein Rps1-k-2 [*Glycine max*] | 4e-34 | Defence | ++ | + | ++ | ++ | + | + | NC | NC |
| FJ226755 | Photosystem II type I chlorophyll a/b-binding protein [*Glycine max*] | 2e-38 | Photosynthesis | + | NC | ++++ | ++ | ++ | - | - | - |
| FJ581436 | rac GTPase activating protein 1 [*Lotus japonicus*] | 2e-24 | Defence | ++ | + | + | + | + | - | + | - |
| GQ922055 | Serine/threonine-protein kinase [*Ricinus communis*] | 7e-58 | Signal transduction | +++ | + | ++ | ++ | ++ | + | + | - |
| FJ621571 | Cysteine protease Cp5 [*Vitis vinifera*] | 1e-61 | Defence | ++ | ++ | - | + | - | - | - | - |
| GU011970 | Zinc finger protein, putative [*Ricinus communis*] | 6e-44 | Signal transduction | +++ | ++ | ++ | + | ++ | ++ | +++ | ++ |
| FJ581437 | LRR -receptor like kinase, putative [*Ricinus communis*] | 6e-99 | Signal transduction | + | NC | - | + | ++ | NC | - | NC |
| GU592825 | Protein kinase -6 [*Glycine max* ] | 2e-23 | Signal transduction | ++ | + | + | + | NC | - | NC | + |
| GQ922056 | Cytochrome P450 monooxygenase CYP97C10 [*Glycine max*] | 2e-125 | Defence | - | - | + | + | NC | - | NC | - |
| GU223575 | Late embryogenesis abundant protein Lea14-A, putative [*Ricinus communis*] | 6e-11 | Abiotic stress | +++ | NC | NC | NC | + | NC | NC | NC |
| GU576549 | Peroxisomal fatty acid β-oxidation multifunctional protein [*Glycine max*] | 9e-42 | Lipid metabolism | ++ | - | - | - | ++ | ++ | - | + |
| GU011971 | Sedoheptulose-bisphosphatase [*Arabidopsis thaliana*] | 2e-28 | Metabolism | ++ | ++ | +++ | + | + | ++ | - | - |
| GQ922058 | Dihydroflavonol-4-reductase [*Medicago truncatula*] | 8e-62 | Secondary metabolite | +++ | +++ | ++ | +++ | +++ | ++ | ++ | ++ |
| GQ922059 | Heat shock 70 kDa protein, mitochondrial-like [*Glycine max*] | 6e-89 | Defence | +++ | + | + | ++ | + | + | NC | - |
| GU576554 | Glyceraldehyde-3-phosphate dehydrogenase [*Pisum sativum*] | 1e-26 | Metabolism | ++ | + | ++++ | - | + | - | +++ | - |
| EU935214 | Oxygen-evolving complex [*Arabidopsis thaliana*] | 2e-57 | Photosynthesis | - | - | - | ++ | - | - | - | - |
| GU326970 | F-box family protein [*Populus trichocarpa*] | 6e-06 | Signal transduction | + | + | + | ++ | ++ | NC | NC | + |
