# Supplementary material for: Transcriptomic and Proteomic Analyses of Resistant Host Responses in Arachis diogoi Challenged with Late Leaf Spot Pathogen, Phaeoisariopsis personata
Source: PLoS One. 2015 Feb 3;10(2):e0117559. doi: 10.1371/journal.pone.0117559 (PMC4315434; doi:10.1371/journal.pone.0117559)
Supplement: S3 Table — (DOCX) [file pone.0117559.s005.docx]

**TDF’s Submitted to GenBank**

| **TDF** | **Accession No.** | **Significant similarity** |
| --- | --- | --- |
| 1. | EU935214 | Thylakoid lumen protein, chloroplast precursor |
| 2. | EU935215 | Cystatin |
| 3. | EU935216 | Adenosine 5'-phosphosulfate reductase |
| 4. | FJ226754 | CLPX; ATP binding / ATPase/ nucleoside-triphosphatase protein |
| 5. | FJ226755 | Photosystem II type I chlorophyll a/b-binding protein |
| 6. | FJ231266 | Multicopper oxidase, putative |
| 7. | FJ231267 | Nodulin 26-like protein |
| 8. | FJ231268 | Methionine synthase |
| 9. | FJ231269 | Hypothetical protein |
| 10. | FJ262533 | CC-NB-LRR type disease resistance protein Rps1-k-1 |
| 11. | FJ262534 | SOF1 protein-like protein [Arabidopsis thaliana] |
| 12. | FJ262535 | Mucin-associated surface protein (MASP) |
| 13. | FJ262536 | Conserved hypothetical protein |
| 14. | FJ581436 | rac GTPase activating protein 1 |
| 15. | FJ581437 | Receptor kinase, putative |
| 16. | FJ621571 | Similar to cysteine protease Cp5 |
| 17. | FJ621572 | Polygalacturonase precursor |
| 18. | FJ621573 | Hypothetical protein |
| 19. | GQ293093 | AAA ATPase; ABC transporter, transmembrane region, type 1 |
| 20. | GQ293094 | Hypothetical protein |
| 21. | GQ293095 | Transcriptional regulator, LysR family |
| 22. | GQ466606 | Glutathione-regulated potassium-efflux system protein, putative |
| 23. | GQ466607 | Thaumatin-like protein 1a-like |
| 24. | GQ922055 | Serine/threonine-protein kinase PBS1, putative |
| 25. | GQ922056 | Cytochrome P450 monooxygenase CYP97C10 |
| 26. | GQ922057 | SGT1-2 |
| 27. | GQ922058 | Dihydroflavonol-4-reductase |
| 28. | GQ922059 | Heat shock 70 kDa protein, mitochondrial-like |
| 29. | GQ979704 | Mg chelatase subunit (46 kD) |
| 30. | GQ979705 | Phytochrome A |
| 31. | GQ979706 | Nucleic acid binding protein, putative |
| 32. | GU011969 | Ribonucleoprotein, chloroplast, putative |
| 33. | GU011970 | Zinc finger protein, putative |
| 34. | GU011971 | Sedoheptulose-bisphosphatase precursor |
| 35. | GU062402 | Valine--tRNA ligase-like protein |
| 36. | GU062403 | Protein alx, putative |
| 37. | GU062404 | Cytochrome c biogenesis |
| 38. | GU062405 | Glycine-rich protein |
| 39. | GU062406 | Sister chromatid cohesion 1 protein, putative |
| 40. | GU133623 | Hypothetical protein |
| 41. | GU223572 | Isoamyl acetate-hydrolyzing esterase, putative |
| 42. | GU223573 | UNC93 homolog A , related |
| 43. | GU223574 | Squamosa promoter-binding protein (SPL1-related), putative |
| 44. | GU223575 | Late embryogenesis abundant protein Lea14-A, putative |
| 45. | GU223576 | Similar to beta-glucosidases |
| 46. | GU223577 | Nucleotide binding protein, putative |
| 47. | GU223578 | Exostosin-like |
| 48. | GU223579 | PRH26 |
| 49. | GU320766 | DNA-binding SAP; Zinc finger, MIZ-type; Zinc finger, FYVE/PHD-type |
| 50. | GU320767 | Putative beta-galactosidase |
| 51. | GU320768 | Putative mutator sub-class protein |
| 52. | GU320769 | Hypothetical protein |
| 53. | GU320770 | P-rich protein NtEIG-C29 |
| 54. | GU320771 | Amine oxidase, putative |
| 55. | GU320772 | U3 small nucleolar RNA (U3 snorna) associated protein, putative |
| 56. | GU320773 | ATP-dependent RNA helicase eIF4A, putative |
| 57. | GU320774 | Unknown Protein |
| 58. | GU326965 | Retrotransposon protein, putative |
| 59. | GU326966 | Unknown Protein |
| 60. | GU326967 | Conserved hypothetical protein |
| 61. | GU326968 | Gag-pol polyprotein |
| 62. | GU326969 | Endo beta n-acetylglucosaminidase, putative |
| 63. | GU326970 | F-box family protein |
| 64. | GU326971 | Polyprotein |
| 65. | GU326972 | Retrotransposon gag protein |
| 66. | GU326973 | Conserved hypothetical protein |
| 67. | GU473162 | Unnamed protein product |
| 68. | GU473163 | Conserved hypothetical protein CBG02792 |
| 69. | GU473164 | Hypothetical protein LOC100254775 |
| 70. | GU473165 | Hypothetical protein |
| 71. | GU473166 | Protein kinase, putative |
| 72. | GU473167 | Squamosa promoter-binding protein, putative |
| 73. | GU473168 | Hypothetical protein |
| 74. | GU473169 | Probable NADH dehydrogenase-like |
| 75. | GU473170 | Cellulose synthase catalytic subunit |
| 76. | GU473171 | Microtubule-associated protein, putative |
| 77. | GU576546 | Conserved hypothetical protein |
| 78. | GU576547 | GIGANTEA |
| 79. | GU576548 | Hypothetical protein |
| 80. | GU576549 | Peroxisomal fatty acid beta-oxidation multifunctional protein |
| 81. | GU576550 | Hypothetical protein |
| 82. | GU576551 | ATP/ADP transporter |
| 83. | GU576552 | Hypothetical protein |
| 84. | GU576553 | Putative mutator sub-class protein |
| 85. | GU576554 | NADP-dependent glyceraldehyde-3-phosphate dehydrogenase-like |
| 86. | GU576555 | Hypothetical protein DEHA0G25157g |
| 87. | GU592818 | Phytochrome A1 |
| 88. | GU592819 | SWI/SNF complex subunit Snf59 |
| 89. | GU592820 | NB-LRR type disease resistance protein Rps1-k-2 |
| 90. | GU592821 | Pentatricopeptide repeat-containing protein, putative |
| 91. | GU592822 | Nucleobase ascorbate transporter |
| 92. | GU592823 | Nucleic acid binding protein, putative |
| 93. | GU592824 | Hypothetical protein |
| 94. | GU592825 | Protein kinase (PK) |
| 95. | GU592826 | Granule-bound glycogen synthase |
| 96. | GU592827 | DNAJ heat shock N-terminal domain-containing protein |
| 97. | GU785012 | Transposon protein |
| 98. | GU785013 | Cytochrome P450 monooxygenase CYP76E3 |
| 99. | GU785014 | N-alpha-acetyltransferase, NatC auxiliary subunit-like |
| 100. | GU785015 | Always early, putative |
| 101. | GU785016 | Unknown protein |
| 102. | GU785017 | Short-chain dehydrogenase/reductase |
| 103. | GU785018 | NADPH oxidoreductase homolog |
| 104. | GU785019 | Gag-pol polyprotein |
| 105. | GU785020 | HAT dimerization protein |
| 106. | GU785021 | Unknown protein |
| 107. | GU785022 | Predicted protein |
| 108. | GU785023 | Hypothetical protein |
| 109. | GU133623 | Hypothetical protein |
| 110. | JZ356596 | No significant similarity |
| 111. | JZ356597 | tRNA dimethylallyltransferase 9-like |
| 112. | JZ356598 | 14-3-3-like protein |
| 113. | JZ356599 | Unknown protein |
| 114. | JZ356600 | Fas-binding factor 1 |
| 115. | JZ356601 | ATP-dependent DNA helicase PIF1 |
| 116. | JZ356602 | Unknown protein |
| 117. | JZ356603 | Unknown protein |
| 118. | JZ356604 | Helicase swr1 |
| 119. | JZ356605 | Hypothetical protein |
| 120. | JZ356606 | Toll-like receptor 6 |
| 121. | JZ356607 | Unknown protein |
| 122. | JZ356608 | Unknown protein |
| 123. | JZ356609 | Unknown protein |
| 124. | JZ356610 | Unknown protein |
| 125. | JZ356611 | RNA-directed DNA polymerase homolog T13L16.7 |
| 126. | JZ356612 | Unknown protein |
| 127. | JZ356613 | Hypothetical protein |
| 128. | JZ356614 | Unknown protein |
| 129. | JZ356615 | Unknown protein |
| 130. | JZ356616 | Hypothetical protein |
| 131. | JZ356617 | Unknown protein |
| 132. | JZ356618 | Unknown protein |
| 133. | JZ356619 | nbs-lrr resistance protein |
| 134. | JZ356620 | Reverse transcriptase |
| 135. | JZ356621 | RNA-directed DNA polymerase |
| 136. | JZ356622 | Unknown protein |
| 137. | JZ356623 | Unknown protein |
| 138. | JZ356624 | Unknown protein |
| 139. | JZ356625 | Unknown protein |
| 140. | JZ356626 | Unknown protein |
| 141. | JZ356627 | Unknown protein |
| 142. | JZ356628 | Unknown protein |
| 143. | JZ356629 | UDP-glycosyltransferase 83A1-like |
| 144. | JZ356630 | Unknown protein |
| 145. | JZ356631 | Unknown protein |
| 146. | JZ356632 | Helicase swr1 |
| 147. | JZ356633 | Unknown protein |
| 148. | JZ356634 | DNA mismatch repair protein |
| 149. | JZ356635 | Glycoside hydrolase 15-related |
| 150. | JZ356636 | Unknown protein |
| 151. | JZ356637 | Unknown protein |
| 152. | JZ356638 | Unknown protein |
| 153. | JZ356639 | Vacuolar protein sorting 13 homolog A |
| 154. | JZ356640 | Chloroplast magnesium chelatase I subunit |
| 155. | JZ356641 | Unknown protein |
| 156. | JZ356642 | WD repeat-containing protein 48-like |
| 157. | JZ356643 | Hypothetical protein MTR-5g079810 |
| 158. | JZ356644 | Hypothetical protein |
| 159. | JZ356645 | Seryl-tRNA synthetase |
| 160. | JZ356646 | Unknown protein |
| 161. | JZ356647 | Phytochrome |
| 162. | JZ356648 | Unknown protein |
| 163. | JZ356649 | Alanyl-tRNA synthetase |
| 164. | JZ356650 | Unknown protein |
| 165. | JZ356651 | Unknown protein |
| 166. | JZ356652 | Unknown protein |
| 167. | JZ356653 | Unknown protein |
| 168. | JZ356654 | Unknown protein |
| 169. | JZ356655 | Unknown protein |
| 170. | JZ356656 | Unknown protein |
| 171. | JZ356657 | Unknown protein |
| 172. | JZ356658 | Unknown protein |
| 173. | JZ356659 | Unknown protein |
| 174. | JZ356660 | Tyrosine decarboxylase |
| 175. | JZ356661 | Putative decarboxylase |
| 176. | JZ356662 | Hypothetical protein SclubJA-17231 |
| 177. | JZ356663 | Unknown protein |
| 178. | JZ356664 | Unknown protein |
| 179. | JZ356665 | Plastidic ATP/ADP-transporter |
| 180. | JZ356666 | Mg chelatase subunit (46 kD) |
| 182. | JZ356667 | Unknown protein |
| 183. | JZ356668 | Hypothetical protein |
| 184. | JZ356669 | Putative conjugal transfer protein TrbG |
| 185. | JZ356670 | Binding-protein-dependent transport systems inner membrane component |
